# Seasonal and sexual variation in mRNA expression of selected adipokine genes affecting fat deposition and metabolism of the emu (*Dromaius novaehollandiae*)

**DOI:** 10.1038/s41598-022-10232-w

**Published:** 2022-04-15

**Authors:** Ji Eun Kim, Darin C. Bennett, Kristina Wright, Kimberly M. Cheng

**Affiliations:** 1 Faculty of Land and Food Systems, Avian Research Centre, University of British Columbia, 2357 Main Mall, Vancouver, BC V6T 1Z4 Canada; 2 Canada’s Michael Smith Genome Sciences Centre, BC Cancer Research Institute, 570 West 7th Avenue, Vancouver, BC V5Z 4S6 Canada; 3 Present Address: Animal Science Department, California Polytechnic State University, San Luis Obispo, CA 93407 USA

**Keywords:** Evolution, Genetics, Molecular biology, Zoology

## Abstract

Emus are farmed for fat production. Oil rendered from their back and abdominal fat pads has good anti-oxidant and anti-inflammatory properties and has ingredients that promote cell growth. Our objective is to examine the mRNA expression of 7 emu adipokine genes (*eFABP4*, *eSCD1*, *eAdipoQ*, *eAdipoR1*, *eAdipoR2*, *eLEP* and *eLepR*) to identify gene markers that may help improve emu fat production. Back and abdominal fat tissues from 11 adult emus were biopsied at four time points (April, June, August and November). Total RNA was isolated and cDNA was synthesized. Gene specific primers were designed for partial cloning fragments to amplify the open reading frame of the 7 genes. e*LEP* was not expressed in emu fat tissue. Nucleotides and amino acids sequences of the 6 expressed gene were compared with homologs from other species and phylogenetic relationships established. Seasonal mRNA expression of each gene was assessed by quantitative RT-PCR and differential expression analysed by the 2^−ΔΔ*C*^_T_ method. The 6 expressed genes showed seasonal variation in expression and showed association of expression level with back fat adiposity. More whole-genome scanning studies are needed to develop novel molecular markers that can be applied to improve fat production in emus.

## Introduction

Emu (*Dromaius novaehollandiae*) is indigenous to Australia and is the second largest living ratite bird. Australian aborigines first used emu oil for wound healing and pain‐alleviation. Currently emu is farmed globally. In Canada, emu is primarily farmed to produce oil. Emu oil is rendered from both the subcutaneous and retroperitoneal fat tissues^1^ and has anti‐inflammatory and antioxidant formulation with reparative properties^2–5^. Topical application of emu oil has been shown to reduce inflammation associated with reduced levels of interleukin 1-alpha (IL-1α), tumor necrosis factor-alpha (TNFα) and other proinflammatory cytokines in a croton-oil-induced inflammation mouse model^6,7^. More recently, well‐controlled pre‐clinical studies have demonstrated the efficacy of orally‐administered emu oil in attenuating inflammatory intestinal disorders^8–13^. Emu oil has not been approved as a drug for human use but is widely used in veterinary medicine and cosmetic and skincare products.

Emus have seasonal pattern of foraging and fat deposition. Adult emus start to gain fat in spring and summer in preparation for breeding in winter. Like other ratites, emus have paternal incubation and brooding. During the incubation period, males have little feed intake and are sustained by the energy in their stored fat, which amounts to about 10 kg.

Adipose tissue has been recognized not only as a fat storage site but also as an important endocrine organ, affecting systemic energy homeostasis, inflammatory processes and development of insulin resistance^14^. In adipose tissue lipid metabolism, four essential major enzymes and hormones are involved; (1) fatty acid binding protein (FABP4), a soluble protein in the cytoplasm; (2) stearoyl-CoA desaturase (SCD1), a key enzyme that regulates the synthesis of unsaturated fatty acids^15^; (3) adiponectin (AdipoQ), an adipokine hormone that is mainly secreted from mammalian adipose tissue, is involved with lipogenesis and insulin resistance^16^. In birds, adiponectin receptors are expressed in a diversity of tissues and its function may be altered from that of mammals^17–19^; (4) leptin, an adipocyte-derived hormone that regulates feeding behavior in mammals where energy expenditure via its interaction with the leptin receptor (LepR) belongs to the class I cytokine receptor superfamily^20^.

The FABPs are abundant intracellular proteins that play important roles in the transportation and metabolism of long-chain fatty acids^14,21,22^. FABP family proteins could be used as tissue specific injury markers because they have high tissue specificity, abundance in the tissue, and low molecular weight (approx. 15 kDa)^21,23^. The development and growth of adipose tissue are due to increases of both adipocyte cell size and cell number. The FABP4 has been extensively used as a marker for differentiated adipocytes^14,24^.

AdipoQ is an adipokine hormone that influences several metabolic functions including glucose utilization, lipogenesis, energy homeostasis and immunity by signaling through two distinct receptors, AdipoR1 and AdipoR2. *AdipoR1* is abundantly expressed in skeletal muscle, whereas *AdipoR2* is predominantly expressed in the liver. *AdipoR1* and *AdipoR2* genes are ubiquitously expressed in chicken tissues and their expression is altered by feed deprivation in the anterior pituitary gland and adipose tissue^25^.

Leptin (LEP) is an adipokine hormone that is the central mediator in a negative feedback loop that regulates energy homeostasis through the hypothalamus. In mammals, LEP administration leads to reduced food intake, increased energy expenditure and weight loss^26^. Unlike in mammals, *LEP* shows no expression in adipose tissue of the few avian species examined so far^27,28^, while its receptor (*LepR*) is still weakly expressed with no correlation to adiposity in chickens^29^. Previous studies revealed that avian LepR shares signal transduction pathway via administration of mammalian LEP^30^.

In this study, we first cloned these adipokine genes, *FABP4*, *SCD1*, *AdipoQ*, *AdipoR1*, *AdipoR2*, *and LepR*, from emu adipose tissue and investigated the seasonal gene expression profile associated fat deposition with the intention to identify genetic markers for improving fat production in the emu.

We tested the following null hypotheses:

### H_0_ 1

The selected emu adipokine genes do not share DNA and amino acid sequence similarity with homologous genes in other avian species.

### H_0_ 2

In emus, the selected adipokine genes have no seasonal variation in expression between April and November.

### H_0_ 3

In emus, the selected adipokine genes are not associated with back and abdominal fat adiposity.

### H_0_ 4

In emus, *LEP* gene expression can be detected in the back and abdominal fat pads.

### H_0_ 5

In emus, the selected adipokine genes are not associated with fatty acids profile in November.

### H_0_ 6

In emus, there is no difference between males and females in their fat pad fatty acids profile in November.

## Methods

The study was conducted in accordance with the relevant guidelines and regulations. Methods are reported in accordance with ARRIVE guidelines (https://arriveguidelines.org).

### Animal tissue

We tracked 11 adult emus (7 males, 4 females) over one breeding season (TryHarder farm, Saskatchewan, Canada). Birds were weighed and back fat and abdominal fat tissues were biopsied with a tissue punch with plunger (diameter core size: 6.0 mm, TED PELLA Ltd.) at four time points (April, June, August and November 2011). These time points were chosen as April was the time when birds started to put on fat, June was the time when birds were gaining fat, August was the time when maximum fat was put on and between August and November there were minimum fat gain, November was the start of the breeding season and birds started to use back fat as an energy source. In addition, for June, August and November, 6 birds from the same flock were also sampled. Different 6 birds were sampled for each of these 3 time points (See Supplemental Table S1). A total of 62 samples were collected. The samples were kept in RNALater (Ambion, Carlsbad, CA) at -20 °C until use. In November 2011, the birds were slaughtered and the back and abdominal fat and body weight were recorded. Emu fat was rendered into oil (see “Fatty acids analysis” section below). All studies were approved by the Animal Care and Use Committee at University of British Columbia (Certificate # A10-0106).

### Total RNA extraction and cloning

Adipose tissue (0.2 g) in RNALater^®^ was used to isolate total RNA with TRI Reagent (Sigma, St. Louis, MO) and RNeasy kit (Qiagen, Toronto, Ontario) using TRI Reagent^31^. Total RNA was quantified on a NanoDrop 2000 (Thermo Scientific, Wilmington, DE). The first strand cDNA was synthesized using SuperScript First-Strand Synthesis System (Invitrogen, Carlsbad, CA) and followed the manufacture protocol. Based on EST database of other avian species (mainly *Gallus gallus*, *Meleagris gallopavo*, *Anser anser*, *Taeniopygia guttata*), the gene specific primers were designed and used for partial cloning fragments to amplify the open reading frame of *FABP4*, *SCD1*, *AdipoQ*, *AdipoR1*, *AdipoR2*,* LepR* and *β-actin* (Table 1). Because of the uncertainty of *LEP* expression in avian adipose tissue, we have designed specific primers based on the conserved *LEP* gene region of six different species (*Gallus gallus*, *Meleagris gallopavo*, *Anas platyrhynchos*, *Silurus asotus*, *Mus musculus*) for amplifying *LEP* mRNA in emu adipose tissue (Table 2). Never the less, we were not able to amplify *LEP* mRNA in emu adipose tissue. The PCR was performed using pfuUltra high fidelity DNA polymerase (Stratagene, Mississauga, ON) and the PCR profile was 2 min at 94 °C, 30 s at 94 oC, 30 s at annealing temperature 53–60 °C (25 cycles) and 90 s at 72 °C, followed by a final extension at 72 °C for 10 min. The amplicon of each gene was subcloned into Zero Blunt PCRII vector (Invitrogen, Carlsbad, CA) and sequenced at NAPS Unit, University of British Columbia. Sequence data were analyzed using Lasergene SeqManII software (DNASTAR Inc., Madison, WI, https://www.dnastar.com/software/). The final sequence was confirmed by at least three clones in any segment, with at least two sequenced from either direction.

**Table 1:** Primers used for amplifying *eFABP4*, *eSCD1*, *eAdopoQ*, *eAdipoR1*, *eAdipoR2*, *eLepR and eβ-actin* from emu adipose tissue.

Gene*	Primer sets	GenBank accession number (ORF^a^)
*eFABP4*	FABP _F1: 5′-GCCTGACAAAATGTGCGAC-3′	JN663389 (399 bp)
	FABP_R1: 5′-AAGAGTTTACGAAAGAGCATGAGGAA-3′	
*eSCD1*	SCD_F1: 5′-CACATGCCTGCGCACTTGCTACA-3′	JN663390 (1083 bp)
	SCD_R1: 5′-GACTACTCCACCAGTGAGTTTGGCTGGC-3′	
	SCD_F2: 5′-GGAATATCATCCTCATGAGCCTGCTGCA-3′	
	SCD_R2: 5′-TGGGAGTCACAAGAGCGGCTGAGTTC-3′	
*eAdipoQ*	AdipoQ_F1: 5′-ACGTTTACCGCTCCGCCTTCAGCGT -3′	JQ289558.1 (738 bp)
	AdipoQ_R1: 5′- AGGCTGACCTTGACGTCTGACAG-3′	
	AdipoQ_F2: 5′-AACAACGTCGACCAAGCGAGCGGTT-3′	
	AdipoQ_R2: 5′-CCTTTCTCTCCCTTTTGTCCGTCT-3′	
	AdipoQ_F3: 5′-ATGTGGGGCGCAGCCCGCTTC-3′	
	AdipoQ_R3: 5′-TTAGTGGAGATCCAAGTCTGGATAAAG-3′	
*eAdipoR1*	AdipoR1_F1: 5′-ATATGGCGTCCCGGAAAGCCGC-3′	JQ289559.1 (1059 bp)
	AdipoR1_R1: 5′-AGATGCCCAGGACACAAACGATGGA-3′	
	AdipoR1_F2: 5′-TCTTCCGAATACACACCGAGACGG-3′	
	AdipoR1_R2: 5′-TCAGAGGAGAGAGTCATCTGTGCAC-3′	
*eAdipoR2*	AdipoR2_F1: 5′-ATG AATGAACTAACGGAACTCGATAATGC-3′	JQ289560.1 (1158 bp)
	AdipoR2_R1: 5′-TTACTGCATCCCCTCCTCTTCT-3′	
*eLepR*	LepR_F1: 5′-ATGTATCATCAAATCATTCTGACCATGTC-3′	JQ289561 (3456 bp)
	LepR_R1: 5′-GAAGAAATCCCAGAAAGTCAGTATACGC-3′	
	LepR_F2: 5′- AGCACGTGTGTGATTTTGACTTGGAC-3′	
	LepR_R2: 5′-CAGATCAGGTGGGCTTTACGAACAGAA-3′	
	LepR_F3: 5′-AGCACGTGTGTGATTTTGACTTGGAC-3′	
	LepR_R3: 5′-GCAAGAGACCACAGAGAACAGCTGTTAA-3′	
*eβ-actin*	*β-actin*_F: 5′-ATG GATGATGATATTGCTGCG-3′	JN663391 (1128 bp)
	*β-actin*_R: 5′-CCACCGCAAATGCTTCTAA-3′	

*eFABP: emu Fatty Acid Binding Protein*; eSCD1:* emu Stearoyl-CoA desaturase-1*; eAdipoQ:* emu Adiponectin*; eAdipoR1:* emu Adiponectin Receptor1*; eAdipoR2:* emu Adiponectin Receptor2*; eLepR:* emu Leptin Receptor*; eβ-actin:* emu beta-actin.

^a^*ORF* Open reading frame.

**Table 2:** Primers used for amplifying e*Lept* from emu adipose tissue*.

Gene	Primer sets
*eLEP*	LEP_F1: 5′- ATGTGCTGGAGACCCCTGTGTCGA-3′
	LEP_R1: 5′- TCAGCATTCCGGGCTAATATCCAACTG-3′
	LEP_F2: 5′- ATGTGCTGGAGACCCCTGTGTCGACTT-3′
	LEP_R2: 5′- TCAGCATTCCGGGCTAATATCCAACTGTT-3′
	LEP_F3: 5′-CTCATCAAGACCATTGTCACCAGGATC-3′
	LEP_R3: 5′-AGCAGCTCTTGGAGAAGGCCAGCA-3′
	LEP_F4: 5′-CTGAGTTTGTCCAAGATGGACCAGAC -3′
	LEP_R4: 5′- AGCACATTTTGGGAAGGCAGGCTGG-3′
	LEP_F5: 5′-AGACCTCCTCCATCTGCTGGCCTT-3′
	LEP_R5: 5′-GTGAAGCCCAGGAATGAAGTCCAAGC-3′

*Because in the few avian species that have been examined, *LEP* expression was not found in adipose tissue, we have designed primers based on the conserved region of *LEP* from 6 different species (wild mallard, Japanese eel, catfish, mouse, chicken and turkey) to determine whether *LEP* expression can be found in emu adipose tissue.

### Amino acids similarity

Putative amino acid sequences of emu FABP4 (eFABP4), emu SCD1 (eSCD1), emu AdipoQ (eAdipoQ), emu AdipoR1 (eAdipoR1), emu AdipoR2 (eAdipoR2) and emu LepR (eLepR) were aligned with homologs from other species and the sequence similarity of amino acid was compared using ClustalW 2.0 (http://www.ebi.ac.uk/Tools/msa/clustalw2/). Conserved domain within a protein sequence was used NCBI (http://www.ncbi.nlm.nih.gov/Structure/cdd/wrpsb.cgi). PRINTS (http://www.bioinf.man.ac.uk/dbbrowser/PRINTS/index.php).

### Phylogenetic analysis

Amino acid sequences of the eFABP4, eSCD1, eAdipoQ, AdipoR1, AdipoR2 and eLepR of the emu were aligned with those of 12 other species (including 7 other avian species) and compared using the phylogenetic and molecular evolutionary analysis software, MEGA X 10.2 (http://www.megasoftware.net/mega.php)^32^. Bootstrapped neighbor-joining method was used for phylogenetic reconstruction. Five hundred bootstrap replicates were employed. These 12 species were *Anas platyrhynchos* (wild mallard duck, ABC96712.2), *Anser anser* (greylag goose, AAL79836.1), *Anser cygnoides* (swan goose, XP_013028005.1), *Taeniopygia guttata* (zebra finch, XP_002199746.1), *Gallus gallus* (chicken, AAL30743.1), *Meleagris gallopavo* (turkey, XP_003205187.1), *Phasianus colchicus* (pheasant, XP_031446733.1), *Homo sapiens* (human, NP_001433.1), *Mus musculus* (mouse, EDL05171.1), *Salmo salar* (salmon, AGH92578.1), *Anolis carolinensis* (Carolina anole lizard, XP_003219598.1), and *Xenopus tropicalis* (Western clawed frog, NP_001015823.1).

### Quantitative real time PCR

Back adipose tissues of the 11 adult birds in April and 17 adult birds each from June, August, and November were used (Table S1). Total RNA from each adipose sample was extracted using TRI Reagent (Invitrogen, Carlsbad, CA). The first-strand cDNA was synthesized by reverse transcribing total RNA using Oligo(dT)_12–18_ primer, and 2,000U Superscript III reverse transcriptase (Invitrogen, Carlsbad, CA). Primers obtained from GenBank, RACE and partial sequencing (Tables 1 and 2) were used to amplify the specific candidate genes.

For RT-PCR, gene-specific primers (Table 3) were used in PCR reactions to amplify corresponding cDNA sequences under the following PCR conditions: 94 °C for 3 min, followed by 35 cycles of (94 °C for 30 s, 53 °C for 30 s, and 72 °C for 1 min) followed by 72 °C for 10 min, using Taq polymerase in a 50-μL total reaction. Housekeeping gene *eβ*-actin was used as control (Table 1). For quantitative RT-PCR of *eFABP4*, *eSCD1*, *eAdipoQ*, *eAdipoR1*, *eAdipoR2*, and *eLepR* expression, 700 ng of cDNA was incubated with 10 μL iQ SYBR Green Supermix (Bio-Rad, Hercules, CA) and 10 pmol of each forward and reverse primer in a total volume of 20 μL. An initial denaturation step at 95 °C for 2:30 min followed by 40 cycles at 95 °C for 15 s, 53 °C for 15 s, and 72 °C for 30 s. Each qRT-PCR run had technical duplicate samples to generate average quantification cycle data per run (iQ5 Real-Time PCR Detection Systems, Bio-Rad, Canada). At the end of amplification, a melting curve analysis was done by heating the PCR products to 55–95 °C and the fluorescence was detected to confirm a single amplification product. Three biological replicates were averaged for quantitative analysis.

**Table 3:** Gene specific primers for amplifying the specific gene fragment of emu *eFABP*, *eSCD-1*, *eAdipoQ*, *eAdipoR1*, *eAdipoR2*, *eLepR and eβ-actin* for quantitative real time PCR.

Gene	Gene specific primers	Expected size (bp)
*eFABP4*	FABP-RT _F1: 5′-CTGGTGTGGCCAAGCCCA-3′	172
	FABP-RT _R1: 5′-GAGCCATTATCTAGGGTTATG-3′	
*eSCD1*	SCD-RT _F1: 5′-CATCAACCCACGAGAGAACC-3′	223
	SCD-RT _R1: 5′-ATCTCCAGTCCGCATTTTCCG-3′	
*eAdipoQ*	AdipoQ-RT_F1: 5′-ACGTCCCCATCCTATTCAGC-3′	189
	AdipoQ-RT_R1: 5′-GGAACTGGTCGTAGGTGAAGA-3′	
*eAdipoR1*	AdipoR1_RT_F1: 5′-TGCTGCGGCCCAACATGTATT-3′	193
	AdipoR1_RT_ R1: 5′-AAGCTCCCCATGATCAGCAG-3′	
*eAdipoR2*	AdipoR2_RT_F1: 5′-ACGGAACTCGATAATGCTGGTT-3′	242
	AdipoR2_RT_R1: 5′-GCATGGTGGGCTTGTAGAAG-3′	
*eLepR*	LepR-RT_F1: 5′-AGATACTGACCAGTGTTGGTTC-3′	162
	LepR-RT_R1: 5′- GAGTAACTTTGCTTACGCGATC-3′	
*eβ-actin*	***β***-actin-RT_F1: 5′-CTGGCACCTAGCACAATGAA-3′	123
	***β***-actin-RT_R1: 5′-CTGCTTGCTGATCCACATCT-3′	

*eFABP4:* emu Fatty Acid Binding Protein*; eSCD1:* emu Stearoyl-CoA desaturase-1*; eAdipoQ:* emu Adiponectin*; eAdipoR1:* emu Adiponectin Receptor1*; eAdipoR2:* emu Adiponectin Receptor2*; eLepR:* emu Leptin Receptor*; eβ-actin:* emu beta-actin.

### Differential expression

The relative changes in e*FABP4*, e*SCD1*, *eAdipoQ*, *eAdipoR1*, *eAdipoR2*, and *eLepR* expression over time were measured by the relative quantification of their qRT-PCR signal in the 4 time points (April, June, August, and November) and analysed by the 2^−ΔΔ*C*^_T_ method^33^.

### Fatty acids analysis

Fat samples collected in November were thawed, weighed and ground (cold pressed). The samples were then placed in a double boiler pan and heated at 70 °C for 30 min to yield the rendered oil. The resulting oil samples were then filtered, weighed and stored under nitrogen in white plastic bottles at 4 °C.

Total lipids in the oil samples were extracted with chloroform:methanol (2:1 v/v) containing 0.01% butylated hydroxytoluene (BHT) as the antioxidant. Fatty acid methyl esters (FAME) were prepared by transesterification with Boron trifluoride (BF3) in methanol following the method described by Kitts et al.^34^, based on the procedure described by Ackman et al.^35^. After the extraction with hexane, FAMEs were analyzed by gas liquid chromatograph (GC-17A, Shimadzu Scientific Instruments Inc., Columbia, Maryland), equipped with flame ionization detector and an auto injector (AOC1400, Shimadzu Scientific Instruments Inc., Columbia, Maryland). Samples were injected onto a capillary column (30 m × 0.25 mm; 0.25 μm film thickness; liquid phase: J and W DB 23), with helium as the carrier gas. Temperature programming was used according to the method described by Budge et al.^36^ with minor modification. The column temperature was initially set at 153 °C for 2 min, then increased to 174 °C at 2.3 °C/min, and then to a final temperature of 220 °C at 2 °C/min with a final hold time of 2 min. Detector and injector temperatures were both set at 250 °C. Chromatographic peaks were integrated and identified using the Shimadzu software package (version 7.2.1 SP1, https://www.shimadzu.com/an/products/software-informatics/software-option/index.html), which were compared to known standards supplied by Nu-Chek Prep (Elysian, MN). Individual fatty acids are reported as weight percent of total fatty acids using mass response factors relative to C18:0.

### Statistical analysis

Repeated Measures Mixed Model Least Squares ANOVA tests and multiple regression analyses were performed using JMP 8.0 (SAS Institute, Cary, North Carolina, USA). Tukey’s HSD was used for mean separation and the level of significance was defined at *P* < 0.05.

Statistical models:

1. Seasonal variation in gene expression
  $$ Y_{ijk} = \mu + Sex_{i} + e_{ij} + Season_{k} + \, \left( {Season \times Sex} \right)_{ik} + e_{ijk} $$
  where *Y*_*ijkl*_ = the mRNA expression level of an adipokine gene, *μ* = the grand mean, *Sex*_*i*_ = the effect of sex, *i* = male or female, *e*_*ij*_ = the effect of replication and the sub-plot error term for testing the sex effect, *j* = 1, 2, 3…17, *Season*_*k*_ = the effect of season, *k* = April, June, August, or November time point, (*Season x Sex*)_*ik*_ = the interaction between sex and season, *e*_*ijk*_ = model error term.
  Both *Season* and *Sex* and their interaction were treated as fixed terms. *Replication* is considered as a random effect nested in *Sex*.
2. Association between fat gain and level of gene expression
  $$ \begin{aligned} \hat{Y}_{(Apr - June)} & = \beta_{0} + \beta_{1} \left( {eFABP4_{(Apr)} } \right) + \beta_{2} \left( {eSCD1_{(Apr)} } \right) + \beta_{3} \left( {eAdipoQ_{(Apr)} } \right) + \beta_{4} \left( {eAdipoR1_{(Apr)} } \right) \\ & \quad + \beta_{5} \left( {eAdipoR2_{(Apr)} } \right) + \beta_{6} \left( {eLepR_{(Apr)} } \right) \\ \hat{Y}_{(June - Aug)} & = \beta_{0} + \beta_{1} \left( {eFABP4_{(June)} } \right) + \beta_{2} \left( {eSCD1_{(June)} } \right) + \beta_{3} \left( {eAdipoQ_{(June)} } \right) + \beta_{4} \left( {eAdipoR1_{(June)} } \right) \\ & \quad + \beta_{5} \left( {eAdipoR2_{(June)} } \right) + \beta_{6} \left( {eLepR_{(June)} } \right) \\ \hat{Y}_{(Aug - Nov)} & = \beta_{0} + \beta_{1} \left( {eFABP4_{(Aug)} } \right) + \beta_{2} \left( {eSCD1_{(Aug)} } \right) + \beta_{3} \left( {eAdipoQ_{(Aug)} } \right) + \beta_{4} \left( {eAdipoR1_{(Aug)} } \right) \\ & \quad + \beta_{5} \left( {eAdipoR2_{(Aug)} } \right) + \beta_{6} \left( {eLepR_{(Aug)} } \right) \\ \end{aligned} $$
  where $\hat{Y}$ is the is the predicted value of the dependent variable (i.e., amount (kg) of fat gain during the period specified (April–June, June to August, or August to November)); *β*_*0*_ is the value of *Y* when all of the independent variables equal to zero (i.e. the *Y*- intercept); *β*_*1*_*–β6* are the coefficients of the independent variables listed in the equation (*eFABP4*, e*SCD1*, *eAdipoQ*, *eAdipoR1*, *eAdipoR2*, *eLepR* which are the mRNA expression level of the adipokine genes at a month specified (April, June, or August).
3. Association between fatty acid profile and gene expression
  $$ \begin{aligned} \hat{Y} & = \beta_{0} + \beta_{1} \left( {Sex_{(female)} } \right) + \beta_{2} \left( {eFABP4_{(Nov)} } \right) + \beta_{3} \left( {eSCD1_{{\left( {Nov} \right)}} } \right) + \beta_{4} \left( {eAdipoQ_{(Nov)} } \right) + \beta_{5} \left( {eAdipoR1_{(Nov)} } \right) \\ & \quad + \beta_{6} \left( {eAdipoR2_{(Nov)} } \right) + \beta_{7} \left( {eLepR_{(Nov)} } \right) \\ \end{aligned} $$
  where $\hat{Y}$ is *the* predicted value of proportion of a Fatty Acid in the oil extracted from emu fat tissue collected in November; *β*_*0*_ is the value of *Y* when all of the independent variables are equal to zero (i.e. the *Y*- intercept); *β*_*1*_*–β*_*7*_ are the coefficients of the independent variables listed in the equation; *Sex*_(*female*)_ is a one for female birds and zero for male birds; *eFABP4*, e*SCD1*, *eAdipoQ*, *eAdipoR1*, *eAdipoR2*, and *eLepR* are the mRNA expression level of the adipokine genes in November.

### Ethics approval

All experiments were performed in accordance with protocols reviewed and approved by the UBC Animal Care Committee (Certificate # A10-0106).

## Results

### Isolation, amplification and cloning

The full-length cDNA of e*FABP4*, e*SCD1*, e*AdipoQ*, e*AdpoiR1*, e*AdipoR2*, e*LepR*, and *eβ-actin* were isolated and cloned from emu adipose tissue with gene specific primers conserved by other avian species EST database (Table 3). Nomenclature of each gene was based on identities of the primary gene structure to other homologs (Table 1), and was assigned the following GenBank accession numbers: *eFABP4* (JN663389), *eSCD1* (JN663390), *eAdipoQ* (JQ289558.1), *eAdipoR1* (JQ289559.1), *eAdipoR2* (JQ289560.1), *eLepR* (JQ289561), and housekeeping gene *eβ-actin* (JN663391).

Nucleic acid sequence of e*FABP4* (92–94% similarity) and *eAdipoR2* (92–94% similarity), showed the highest similarity with orthologues of other avian species (wild mallard, Greylag goose, Swan goose, zebra finch, chicken, turkey, and pheasant) (Table 4). *eAdipoR1* also showed high similarity (92–94%) with other avian species with the exception of Zebra finch (only 90% similar). *eSCD1 and eAdipoQ* were the next high with 88–91% and 80–85%, respectively. We were not able to amplify any *eLEP* mRNA in emu adipose tissue. e*LepR* showed the most divergent among the 6 genes examined with similarity ranging from 76 (zebra finch) to 88% (Greylag and Swan geese).

**Table 4:** Nucleotide sequences identities and similarities of *FABP4*, *SCD1*, *AdipoQ*, *AdipoR1*, *AdipoR2* and *LepR* between emu (*D. novaehollandiae*) and other animal species*.

(Number of species compared)	*eFABP4* (12)	*eSCD1* (12)	*eAdipoQ* (11)	*eAdipoR1*(11)	*eAdipoR2* (11)	*eLepR* (11)
Species*	(%) identity	(%) identity	(%) identity	(%) identity	(%) identity	(%) identity
Wild mallard	94	91	85	94	93	88
Greylag goose	92	91	85	94	94	88
Swan goose	93	91	85	94	94	88
Zebra finch	92	89	80	90	92	76
Chicken	94	89	82	93	94	85
Turkey	92	88	81	93	94	85
Pheasant	92	88	82	93	94	85
Human	76	71	71	83	81	74
Mouse	76	76	71	84	80	71
Salmon	70	73	67	78	76	44
Carolina anole lizard	77	75	73	87	86	73
Western clawed frog	68	73	–	–	–	–

*Wild mallard (*A. platyrhynchos*), Greylag goose (*A. anser*), Swan goose (*A. cygnoides*), zebra finch (*T. guttata*), chicken (*G. gallus*), turkey (*M. gallopavo*), pheasant (*P. colchicus*), human (*H. sapiens*), mouse (*M. musculus*), salmon (*S. salar*), Carolina anole lizard (*A. carolinensis*), Western clawed frog (*X. tropicalis*).

### Amino acid sequence similarity

The primary protein structure assigned the nomenclature and Genbank accession numbers are: eFABP4 (AET74082), eSCD1 (AET74083), eAdipoQ (AFF19461), eAdopoR1 (AFF19462), eAdipoR2 (AFF19463), eLepR (AFF19464) and eβ-actin (AET74084). The eFABP4 encodes a protein of 132 amino acids and shares high similarities with other avian species (average 96.1%) (Table 5). The eSCD1 encodes a protein of 360 amino acids and also shares high similarities with other avian species (average 94%). eAdipoQ encodes a protein of 245 amino acids and shows average 88.5% similarities with other avian species. eAdipoR1 and eAdipoR2 are the most conservative proteins we have examined in this study. eAdipoR1 encodes a protein of 352 amino acids, shares 100% similarity with Swan goose and an average 89.8% similarities with all other species examined. eAdipoR2 encodes a protein of 385 amino acids and shows an average of 92.3% similarities with all other species. The eLepR encodes 1,151 amino acids and shares 90% similarities with the waterfowl group, 86% similarities with chicken, turkey and pheasant, and 79% with zebra finch. (Table 5).

**Table 5:** Amino Acids sequences identities and similarities of FABP4, SCD1, AdipoQ, AdipoR1, AdipoR2 and LepR between emu (*D. novaehollandiae*) and 12 other animal species*.

(Number of species compared)	FABP (12)	SCD (12)	AdipoQ (12)	AdipoR1(12)	AdipoR2 (12)	LepR (12)
Species*	(%) similarity	(%) similarity	(%) similarity	(%) similarity	(%) similarity	(%) similarity
Wild mallard	95	95	90	84	97	90
Greylag goose	96	94	89	83	97	90
Swan goose	97	94	89	100	97	90
Zebra finch	96	94	86	94	94	79
Chicken	93	93	89	98	98	87
Turkey	98	94	89	87	97	86
Pheasant	98	94	88	98	97	86
Human	84	77	79	84	82	71
Mouse	75	75	82	84	88	69
Salmon	71	83	76	95	80	48
Carolina anole lizard	87	82	79	84	90	72
Western clawed frog	76	85	55	97	91	72

*Wild mallard (*A. platyrhynchos*), Greylag goose (*A. anser*), Swan goose (*A. cygnoides*), zebra finch (*T. guttata*), chicken (*G. gallus*), turkey (*M. gallopavo*), pheasant (*P. colchicus*), human (*H. sapiens*), mouse (*M. musculus*), Salmon (*S. salar*), Carolina anole lizard (*A. carolinensis*), Western Clawed Frog (*X. tropicalis*).

### Phylogeny tree

**Figure 1:**
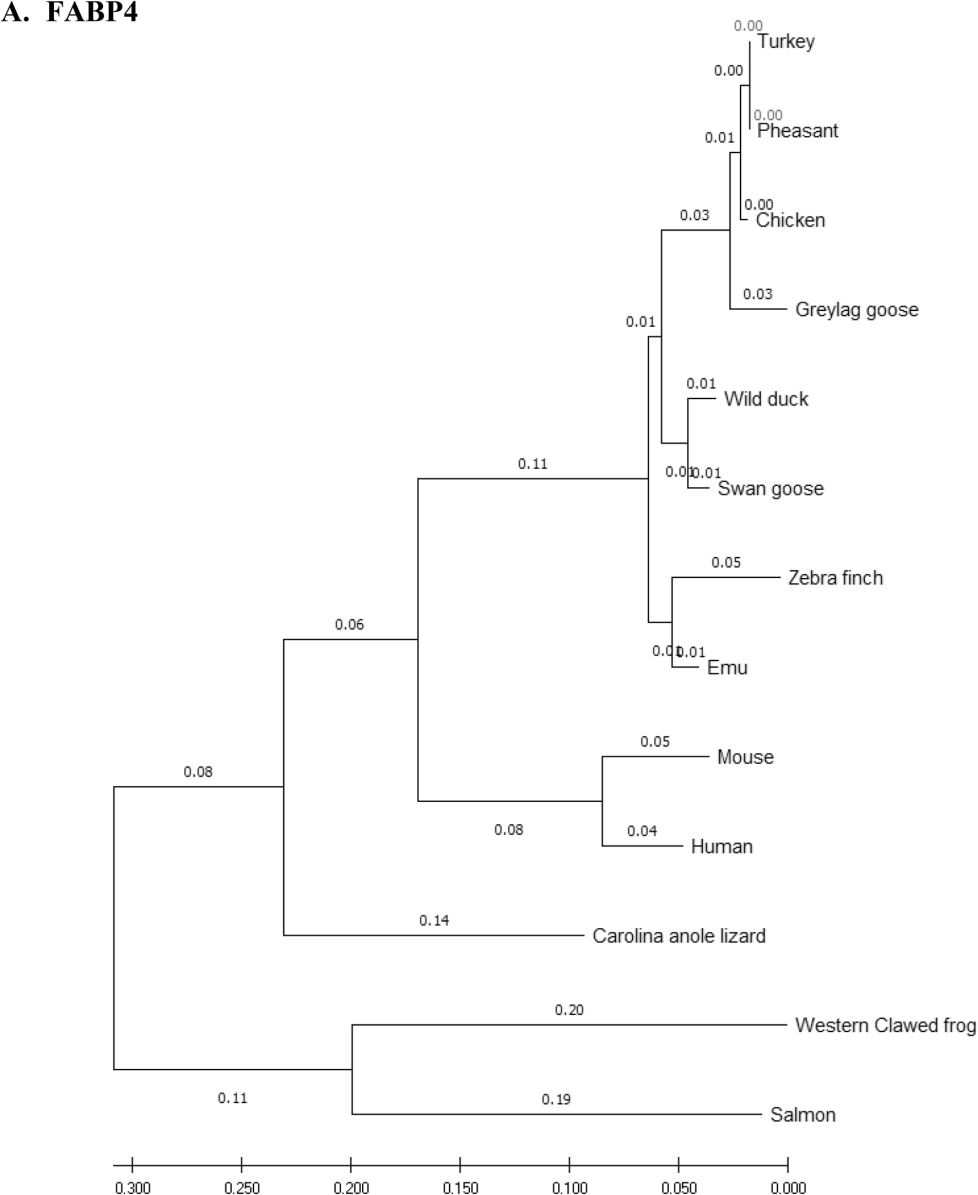

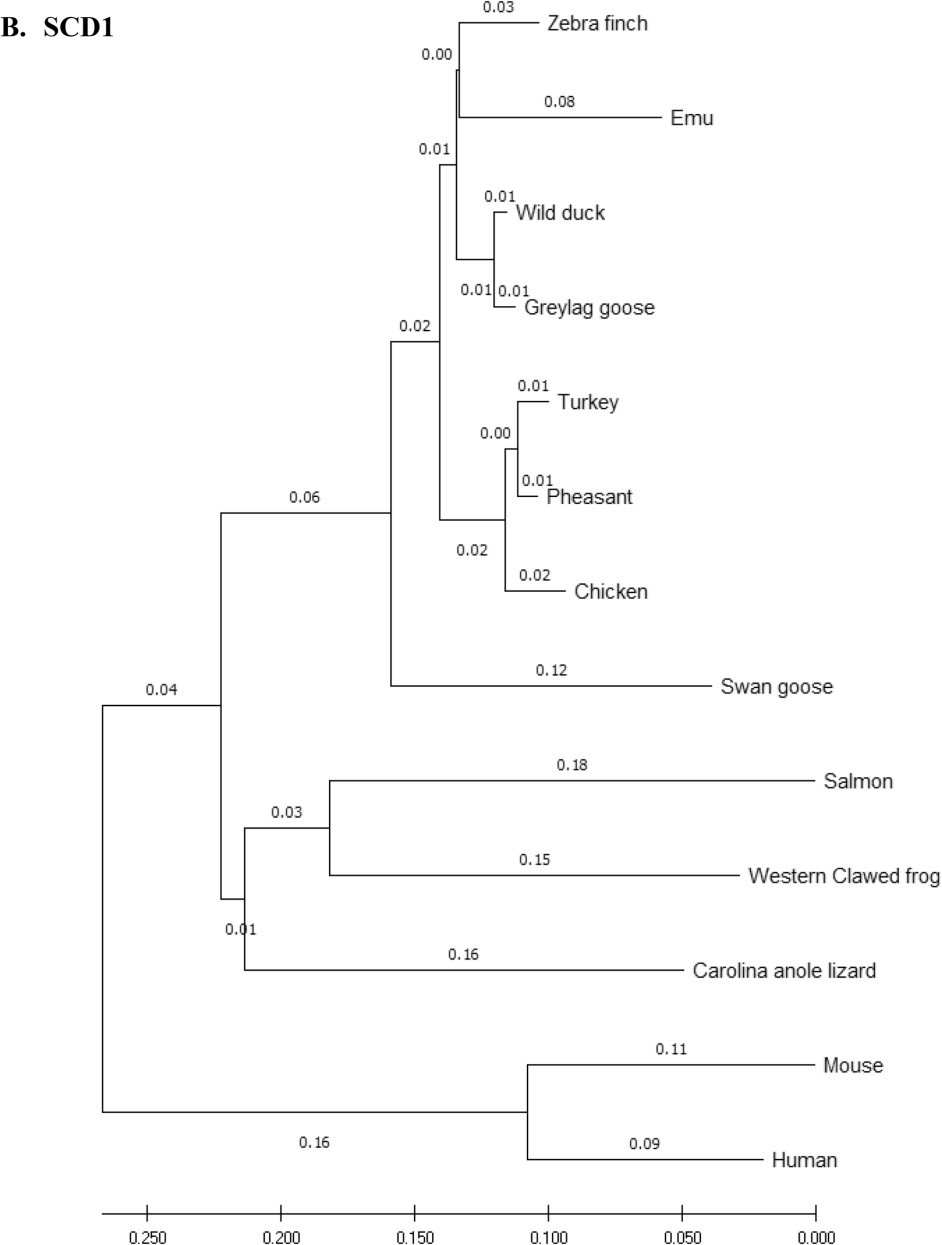

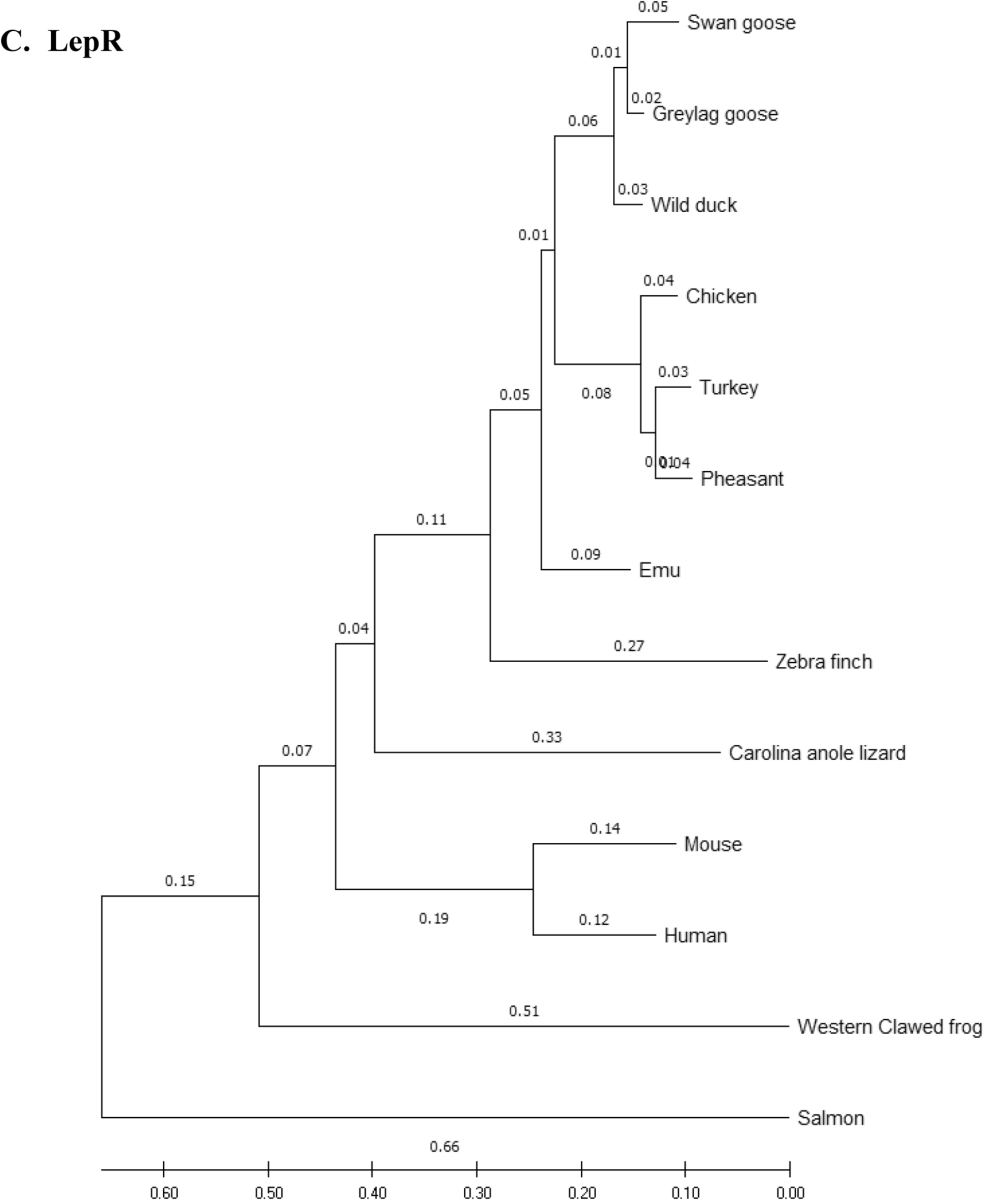

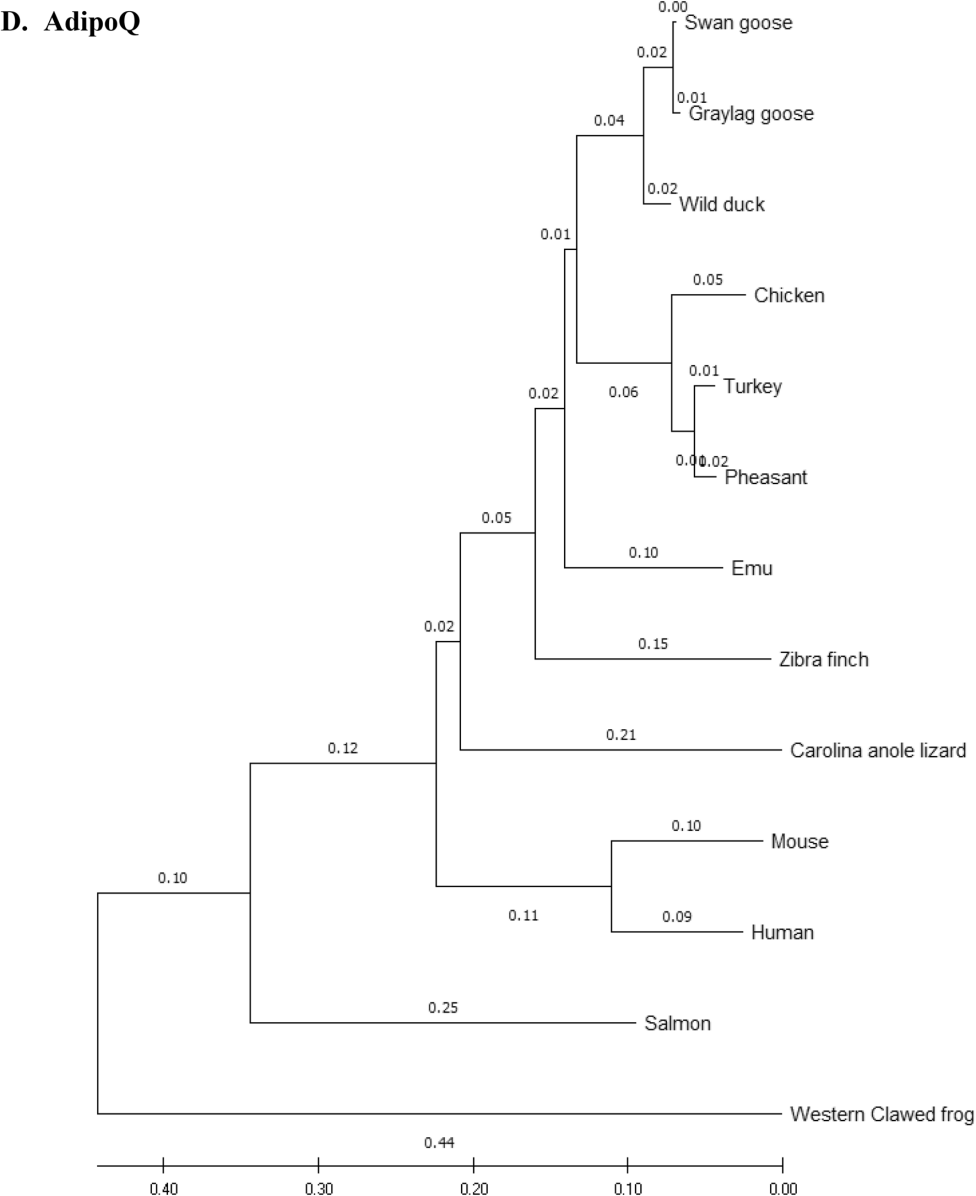

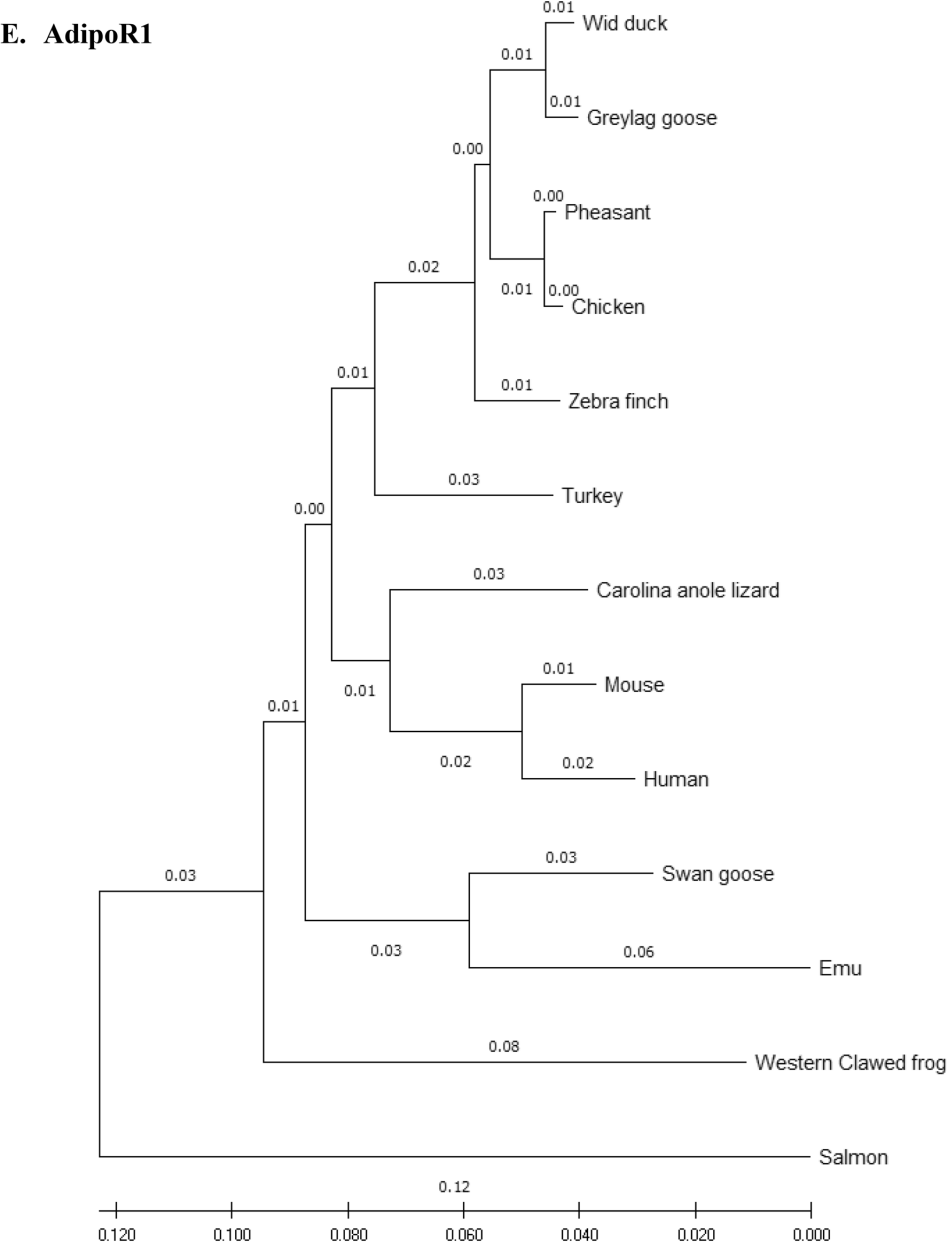

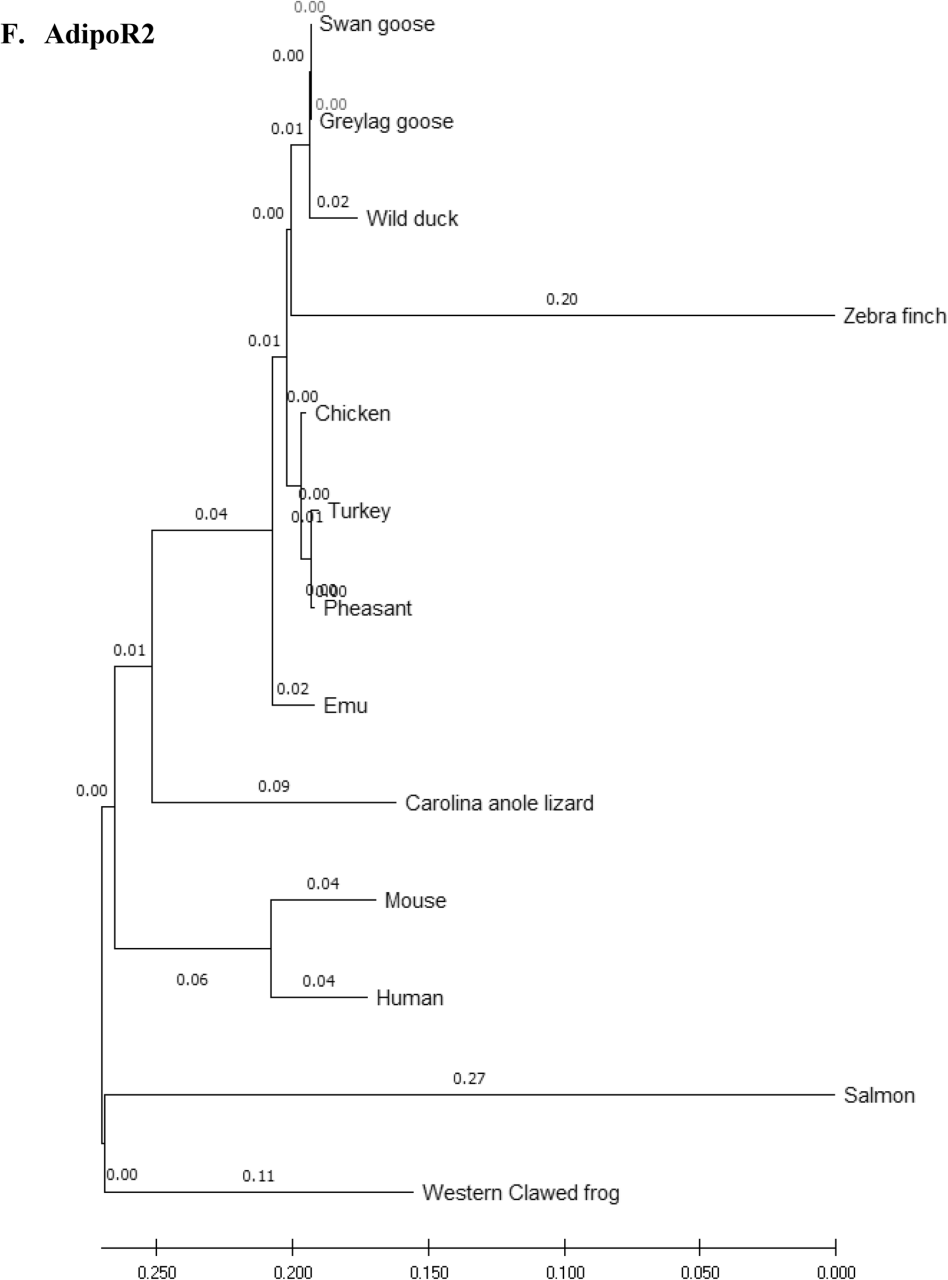
Phylogenetic relationship of emu amino acid sequences with 12 other species: *D. novaehollandiae* (emu, AET74082.1), *Anas platyrhynchos* (wild mallard duck, ABC96712.2), *Anser anser* (greylag goose, AAL79836.1), *Anser cygnoides* (swan goose, XP_013028005.1), *Taeniopygia guttata* (zebra finch, XP_002199746.1), *Gallus gallus domesticus* (chicken, AAL30743.1), *Meleagris gallopavo* (turkey, XP_003205187.1), *Phasianus colchicus* (pheasant, XP_031446733.1), *Homo sapiens* (human, NP_001433.1), *Mus musculus* (mouse, EDL05171.1), *Salmo salar* (salmon, AGH92578.1), *Anolis carolinensis* (Carolina anole lizard, XP_003219598.1), *Xenopus tropicalis* (Western clawed frog, NP_001015823.1). Phylogenetic trees developed using the neighbour-joining method. The numbers in the phylogram nodes indicate percent bootstrap support for the phylogeny. (**A**) FABP4. (**B**) SCD1. (**C**) LepR. (**D**) AdipoQ. (**E**) AdipoR1. (**F**) AdipoR2.

Phylogeny trees of the eFABP1, eSCD1, eAdipoQ, eAdipoR1, eAdipoR2 and eLepR in this study in association with other selected species were constructed to illustrate their genetic relatedness (Fig. 1). Generally speaking, clustering of amino acid sequences resulted in trees showing that emu was closer to zebra finch and migrating waterfowl (swan goose, graylag goose, and mallard) than domestic and gallinaceous birds (turkey, chicken and pheasant). The eFABP4 and eSCD1 are less divergent than eAdipoQ. eAdipoR1 and eAdipoR2 are both less divergent than eAdipoQ, while eAdipoR1 is the least divergent of the emu proteins examined. On the other hand, eLepR is the most divergent from the non-avian proteins.

### Seasonal variation in emu back and abdominal fat weight gain

**Figure 2:**
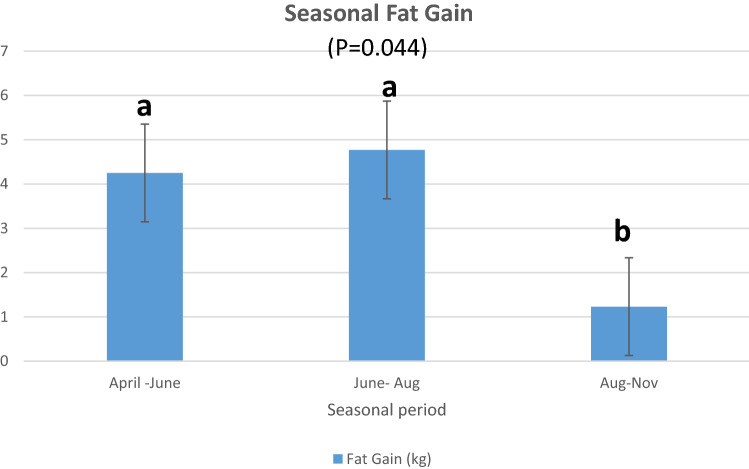
Seasonal variation in emu back and abdominal fat weight gain.

We recorded the body weights of the birds in April, June, August and November. After the birds were slaughtered in November, the back and abdominal fat pads were dissected out and weighed. We also weighed the bird carcasses without the fat pads. The carcass weight (34.8 ± 1.2 kg) was not significantly (*P* < 0.91) different from the April body weight (34.6 ± 1.2 kg). We therefore concluded that the difference in body weight between two time points would be a good estimate of fat gained between the two time points. In this study (Fig. 2), emus gained fat from April to August. From August to November (beginning of breeding season), there was very little fat gain. The mean fat gain from April to November was 11.3 ± 3.28 kg. There was no significant difference between males and females in fat gain.

### Seasonal variation in *eFABP4* expression

**Figure 3:**
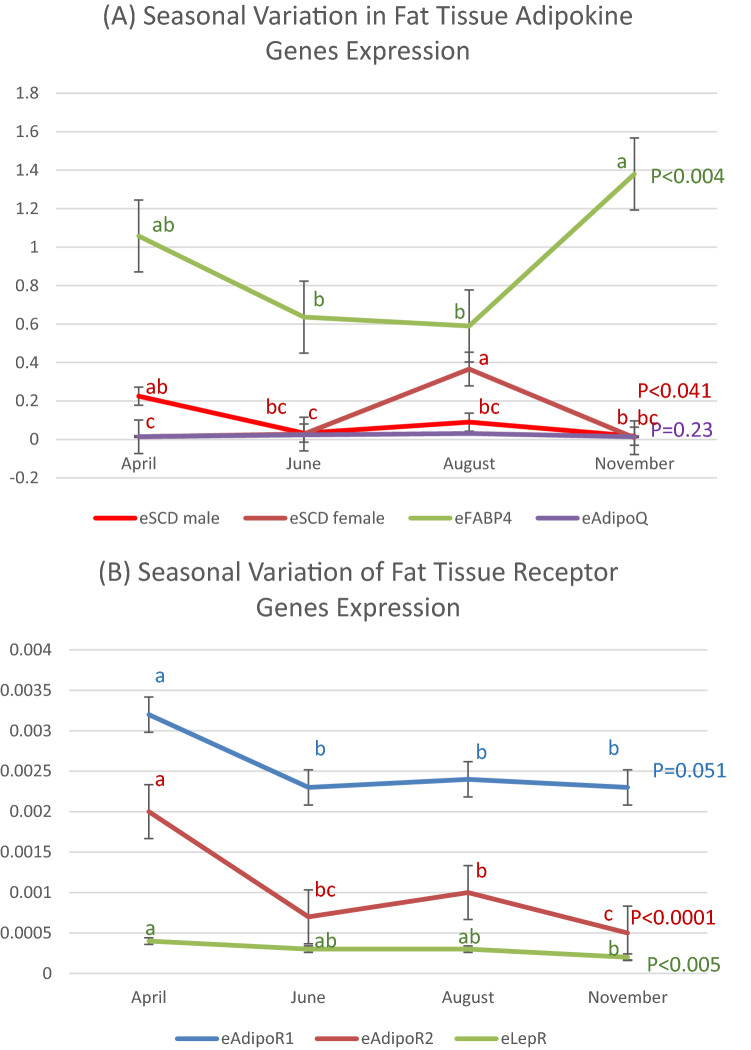
Seasonal variation in mRNA expression (**A**) *eSCD1*, *eFABP4*, *eAdipoQ*, (**B**) *eAdipoR1*, *eAdipoR2*, *eLepR*. *eFABP4:* emu Fatty Acid Binding Protein*; eAdipoQ:* emu Adiponectin*; eAdipoR1:* emu Adiponectin Receptor1*; eAdipoR2:* emu Adiponectin Receptor2*; eLepR:* emu Leptin Receptor.

The level of *eFABP* expression was highest in November, intermediate in April, and low in June and August (Table 6 and Fig. 3A). Sex effect and the Season X Sex interaction were not significant.

**Table 6:** Seasonal variations in mRNA expression levels*.

N = 62	April	June	August	November	*P*
*eFABP4*	1.060 ± 0.208 ab	0.637 ± 0.162 b	0.589 ± 0.162 b	1.379 ± 0.167 a	< 0.004
*eAdipoQ*	0.026 ± 0.004	0.027 ± 0.004	0.030 ± 0.004	0.025 ± 0.004	= 0.23
*eAdipoR1*	0.0031 ± 0.0003 a	0.0023 ± 0.0002 b	0.0024 ± 0.0002 b	0.0023 ± 0.0002 b	= 0.051
*eAdipoR2*	0.0021 ± 0.0002 a	0.0007 ± 0.0001 bc	0.001 ± 0.0001 b	0.0005 ± 0.0001 c	< 0.0001
*eLepR*	0.0004 ± 0.00004 a	0.0003 ± 0.00003 ab	0.0003 ± 0.0003 ab	0.0002 ± 0.0003 b	< 0.005

*eFABP4:* emu Fatty Acid Binding Protein*; eAdipoQ:* emu Adiponectin*; eAdipoR1:* emu Adiponectin Receptor1*; eAdipoR2:* emu Adiponectin Receptor2*; eLepR:* emu Leptin Receptor.

For each gene, means followed by different letters are significantly different by Tukey’s HSD.

*mRNA expression relative to the housekeeping gene *eβ-actin.*

### Regression of fat gained (kg) on *eFABP4* expression levels

The amount of fat gained between April and June regressed significantly and positively on *eFABP4* expression level in April (Table 8 and Supplemental Fig. 1A). The amount of fat gained between June and August regressed significantly and positively on *eFABP4* expression level in June. However, the amount of fat gained between August and November regressed significantly but negatively on *eFABP4* expression level in August.

### Seasonal variation in *eSCD1* expression

There was a significant difference between males and females in their seasonal variation (Season X Sex interaction) in *eSCD1* expression level (Table 7 and Fig. 3A). Male *eSCD1* expression level was slightly higher (not statistically significant) in April and remained low from June to November. Female *eSCD1* level was significantly higher in August but not significantly different than male levels in April, June and November.

**Table 7:** Regression of fat gain on gene expression.

mRNA expression	Fat gain April–June^a^	Fat gain June–August^b^	Fat gain August–November^c^
*eFABP4*	R^2^ = 0.64; *P* = 0.015	R^2^ = 0.94; *P* = 0.0015	R^2^ = **0.71**; *P* = 0.0084
*eSCD1*	*P* = 0.16 ns	R^2^ = 0.97; *P* = 0.0004	*P* = 0.76 ns
*eAdipoQ*	*P* = 0.32 ns	R^2^ = 0.86; *P* = 0.0076	R^2^ = **0.83**; *P* = 0.0017
*eAdipoR1*	R^2^ = **0.55**; *P* = 0.023	R^2^ = 0.92; *P* = 0.0103	*P* = 0.72 ns
*eAdipoR2*	*P* = 0.78 ns	*P* = 0.68 ns	R^2^ = 0.83; *P* = 0.0018
*eLepR*	*P* = 0.20 ns	R^2^ = **0.92**; *P* = 0.0026	*P* = 0.18 ns

*eFABP4:* emu Fatty Acid Binding Protein*; eSCD1:* emu Stearoyl-CoA desaturase-1*; eAdipoQ:* emu Adiponectin*; eAdipoR1:* emu Adiponectin Receptor1*; eAdipoR2:* emu Adiponectin Receptor2*; eLepR:* emu Leptin Receptor.

Bold R^2^ values indicate negative regression.

^a^Fat gain regressed on April mRNA expression.

^b^Fat gain regressed on June mRNA expression.

^c^Fat gain regressed on August mRNA expression.

### Regression of fat gained (kg) on *eSCD1* expression levels

Fat gain was not associated with *eSCD1* expression level except from June to August. The amount of fat gained during that period significantly regressed on June *eSCD1* expression level (Table 8 and Supplemental Fig. 1B).

**Table 8:** Significant (*P* < 0.041) Sex × Season interaction in *eSCD1* expression level.

N = 62	April	June	August	November
Male	0.229 ± 0.08 ab	0.036 ± 0.071 bc	0.090 ± 0.071 bc	0.020 ± 0.067 bc
Female	− 0.082 ± 0.107 c	− 0.021 ± 0.084 c	0.346 ± 0.084 a	− 0.049 ± 0.091 c

Means followed by different letters are significantly different by Tukey’s HSD.

### Seasonal variation in *eAdipoQ* expression

*eAdipoQ* expression level was not significantly (*P* = 0.23) affected by season and there was no significant (*P* = 0.10) difference between males and females (Table 6 and Fig. 3A).

### Regression of fat gained (kg) on *eAdipoQ* expression level

Fat gain from April to June was not associated with *eAdipoQ* expression level. Fat gain from June to August significantly regressed on June *eAdipoQ* level (Table 8 and Supplemental Fig. 1C). Fat gain from August to November significantly but negatively on August *eAdipoQ* level.

### Seasonal variation in *eAdipoR1* expression

*eAdipoR1* expression was significantly higher in April than the rest of the year (Table 6 and Fig. 3B). Sex effect and the Season X Sex interaction were not significant.

### Regression of fat gained (kg) on *eAdipoR1* expression level

The amount of fat gained from April to June significantly but negatively regressed on April *eAdipoR1* level (Table 7 and Supplemental Fig. 1D). From June to August, fat gain regressed significantly on June *eAdipoR1* level.

### Seasonal variation in *eAdipoR2* expression

*eAdipoR2* expression level was highest in April, intermediate in June and August, and lowest in November (Table 6 and Fig. 3B). Sex effect and the Season X Sex interaction were not significant.

### Regression of fat gained (kg) on *eAdipoR2* expression level

Fat gain from April to August was not affected by *eAdipoR2* level. Fat gain from August to November regressed significantly on August *eAdipoR2* level (Table 7 and Supplemental Fig. 1E).

### Seasonal variation in *eLepR* expression

*eLepR* expression level was highest in April, intermediate in June and August, and lowest in November (Table 6 and Fig. 3B). Sex effect and the Season X Sex interaction were not significant.

### Regression of fat gained (kg) on *eLepR* expression level

Amount of fat gained from June to August regressed significantly and negatively on June e*LepR* level (Table 7 and Supplemental Fig. 1F).

### Fatty acid profile

From oil extracted from back and abdominal fat in November, the predominant fatty acids were Oleic Acid and Palmitic Acid (Table 9). Female oil was significantly (*P* < 0.0117) higher in %Palmitic Acid than male oil. Male oil was significantly (*P* < 0.036) higher in %Oleic Acid than female oil. (Table 10 and Supplemental Fig. 2A). Percent Palmitic Acid regress negatively (*P* = 0.043, R^2^ = 0.48) on *eAdipoR1* November expression (Table 10 and Supplemental Fig. 2B). Percent Linoleic acid regressed significantly (*P* = 0.0008) but negatively (R^2^ = 0.54) on November *AdipoR2* level (Table 10 and Supplemental Fig. 2C). Percent α-linolenic acid level also regressed significantly (*P* = 0.0011) but negatively (R^2^ = 0.52) on November *AdipoR2* level (Table 10 and Supplemental Fig. 2D).

**Table 9:** Fatty acid profile of emu fat collected in November.

Fatty acids		Male (N = 14)	Female (N = 9)
C6:0	Caprioic acid	0.16 ± 0.02	0.15 ± 0.03
C8:0^a^	Caprylic acid	0.13	0.16
C12:0^a^	Lauric acid	2.37	0.06
C14:0	Myristic acid	0.38 ± 0.012	0.38 ± 0.016
C14:1n-5	myristoleic acid	0.14 ± 0.014	0.18 ± 0.016
C16:0	Palmitic acid	24.78 ± 0.34	25.97 ± 0.40
C16:1n-7	Palmitoleic acid	5.91 ± 0.36	8.05 ± 0.43
C18:0	Stearic acid	7.29 ± 0.027	6.58 ± 0.032
C18:1n-9	Oleic acid	53.61 ± 0.50	51.89 ± 0.60
C18:1n-7	Cis-Vaccenic acid	0.21 ± 0.023	0.14 ± 0.021
C18:2n-6	Linoleic acid	6.86 ± 0.18	6.46 ± 0.21
C18:3n-3	a-Linolenic acid	0.44 ± 0.03	0.42 ± 0.04
C20:1	Gadoleic acid	0.29 ± 0.04	0.22 ± 0.04
SFA		33.26	33.99
MUFA		59.4	59.13
PUFA		7.31	6.88

^a^Sample size too small for statistical analysis.

**Table 10:** Regression of Fatty Acid levels in emu oil collected in November on sex and November gene expression^a^.

Fatty acids^b^		Sex	*eAdipoR1* expression	*eAdipoR2* expression
C16:0	Palmitic acid	R^2^ = 0.18; *P* = 0.0117	R^2^ = **0.48**; *P* = 0.043	
C18:1n-9	Oleic acid	R^2^ = 0.18; *P* = 0.038		
C18:2n-6	Lenoleic acid			R^2^ = **0.54**; *P* = 0.0008
C18:3n-3	α-Linolenic acid			R^2^ = **0.52**; *P* = 0.0008

^a^Data analysed by mixed model Multiple Regression treating sex as a binary variable (see Statistical Model 3 in “Methods” section). Non-significant variables were removed from the model for final analysis. Bold R^2^ values indicate negative regression. See also Supplemental Fig. 2.

^b^C6:0, C14:0, C14:1n-5, C16:1n-7, C18:0, C18:1n-7, and C20:1 had no significant association with any of the variables in the model. Sample size for C8:0 (N = 4) and C12:0 (N = 2) was too small for statistical analysis.

## Discussion

In order to better understand the genetics of adiposity in emu, a bird that has huge seasonal variation in fat deposition and metabolism, we have selected 7 adipokine genes for examination that have been established to be involved with fat deposition and metabolism. We were able to reject all the null hypotheses that we set out to test. Of the 6 genes (*eFABP4*, *eSCD1*, *eAdipoQ*, *eAdipoR1*, *eAdipoR2*, and *eLepR*) expressed, they shared high nucleotides sequences and amino acids sequences similarity with other avian species (H_0_ 1 rejected). Except for *eAdipoQ*, there were seasonal variation in the mRNA expression between April and November (H_0_ 2 rejected), and the expression level of all 6 genes were associated with back fat weight gain during some time in the season (H_0_ 3 rejected). *eAdipoR1* expression level was associated with the % of Palmitic acid in emu oil while *eAdipoR2* level was associated with % Lenoleic acid and % α-Linolenic Acid in emu oil (H_0_ 5 rejected). There were significant differences between males and females in % Palmitic acid and % Oleic Acid in emu oil (H_0_ 6 rejected).

We were not able to detect any *eLEP* mRNA expression in the emu adipose tissue. Based on the lack of *LEP* expression in the adipose tissue of zebra finch^37^, jungle fowl^38^, several lines of commercial chickens^39^, rock dove^40^, and quail^29^, Friedman and Seroussi^28^ suggested that *LEP* is not expressed in avian adipose tissue. More avian species have to be examined to confirm this observation. Our result from emu, a ratite that is phylogenetically distant from the birds examined so far, provided support to their observations (H_0_4 rejected). In birds, *LEP* is expressed in brain tissue, adrenal glands and gonads, but is not expressed in the liver and is generally not detectable in the blood. *LepR* receptors are predominantly expressed in the pituitary. Seroussi et al.^29^ reported that in chicken, ducks, and quail adipose tissue, *LEP* and *LepR* were scarcely transcribed, and the expression level was not correlated to adiposity. They proposed that LEP in birds may act as an autocrine or paracrine instead of being a circulating hormone as in mammals. These observations, mostly from chicken studies, allowed Friedman and Seroussi^28^ to speculate that avian adipose tissue does not control appetite, insulin resistance, or inflammation.

We have detected low expression levels of *eLepR* in emu fat tissue. Expression level was highest in April and stepwise decreased to the lowest level in November, which was opposite to the fat weight gain trend. The amount of back and retroperitoneal fat gain between June and August regressed significantly but negatively on *eLepR* June expression level. Since there was no *eLEP* expression in emu fat tissue, it seems likely that LEP in emu is still a circulating hormone that affects fat deposition and metabolism. Our phylogenetic analysis found that emu LepR is closer to those of migrating waterfowl than other bird species examined. In mammals, LEP specially repressed the expression of *SCD1* and reduced the accumulation of hepatic triglycerides, cholesterol esters and VLDL synthesis^26,41,42^. It is suspected that the role of LEP in governing adipose tissue regulation of appetite and energy expenditure has been altered in birds^28^. Never the less, the relationship between LEP and the loss of appetite over the winter breeding period in emus remains to be studied.

*eSCD1* is expressed in emu adipose tissue. There was little seasonal variation in expression except that fat samples collected from females in August had a significant 35-fold increase in expression. Fat weight gained between June and August regressed significantly on June *eSCD1* expression level. Individuals that had high fat weight gain would have high *eSCD1* expression but low *eLepR* expression and vice versa for individuals that had little fat weight gain. This would indicate that emu leptin can suppress the expression of *eSCD1* as seen in mammals. SCD1 is also transcriptionally regulated by a number of factors in mammals, including sterol regulatory element-binding protein-1 (SREBP-1) and polyunsaturated fatty acids^43,44^.

SCD1 is predominately located in the endoplasmic reticulum and catalyzes the rate-limiting step in the cellular synthesis of mono-unsaturated fatty acids from saturated fatty acids^15,45^. SCD-1 converts the saturated fatty acids, palmitic acid (16:0) and stearic acid (18:0), to generate the mono-unsaturated fatty acids, palmitoleic (16:1 n7) and oleic acid (18:1 n9), which are accumulated as triglycerides in adipose tissues^45–48^. Oleic acid is the predominant fatty acid in emu adipose tissue. A proper ratio of saturated fatty acids to mono-unsaturated fatty acids contributes to membrane fluidity. In mice, SCD-1, known as a lipid synthesis enzyme, also plays a role in upregulating lipid mobilization through its desaturation product, oleic acid^49^. Specific unsaturated fatty acids are preferentially used during metabolism over saturated fatty acids^50–52^.

Fat storage and usage in birds are mainly for survival, migration and reproductive performance^53–56^. Catbirds increased adipose storage during spring and autumn migration, showing increased rates of basal lipolysis during migration and tropical overwintering^57^. In our study, emus started gaining fat in April and the rate of gain was maximized between June and August. Fat weight gain between June and August significantly regressed on June *eSCD1* expression. From August to November fat gain was minimal. It was during this period when female emus were getting ready to lay eggs. There was a 35-fold increase in *eSCD1* expression in females in August. They may be optimizing the fatty acid composition of the adipose tissue to get ready for the mobilization of lipids into the ovary for formation of the egg yolk. Unfortunately, we only had the fatty acid profile of emu oil collected in November and by that time *eSCD1* expression was extremely low and we found no association of *eSCD1* expression and fatty acid profile of emu oil.

FABPs are a family of proteins known as intracellular lipid chaperones that regulate lipid trafficking and responses in cells^58^. *FABP* genes have been shown to be associated with lipid metabolism (lipogenesis and lipolysis), homeostasis in adipocytes, marbling and back fat deposition^59–61^. *FABP4* is highly expressed in adipocytes and its expression can be highly induced during adipocyte differentiation which is transcriptionally controlled by peroxisome proliferator-activated receptor (PPAR) γ agonists, fatty acids, dexamethasone and insulin^57,62^. It has also been postulated that FABP4 can activate hormone sensitive lipase (HSL) in adipocytes to regulate lipolysis^63,64^. In chickens, earlier studies that examined the relationship of FABP4 with growth and fat accumulation reported results ranging from no association with fat accumulation in hybrid chickens^65^, significant positive correlation with abdominal fat in Luyuan chickens^66^, to correlation with growth depression in Arbor Acre genotype but strong positive association with growth performance of Cobb genotype^61^. In our study, *eFABP4* expression in emu adipose tissue was high both in April and November and relatively low in June and August. Fat gain from April to August regressed positively on April and June *eFABP-4* expression, respectively. However, fat gain from August to November regressed negatively on August *eFABP4* expression. From August to November, fat gain was minimal and a couple of birds even had negative fat gain. By this time of the year, emus started to draw on the energy from the accumulated fat and the role of FABP4 switched from lipogenesis to lipolysis^57^. *eFABP4* expression was highest in November and this may be an indication that the birds were more and more dependent on fat for energy because they have very little feed intake during breeding. In geese, FABP4 was found to be involved in lipid transportation and metabolic process, follicle development and final egg production. *FABP4* was upregulated in the laying group compared with the pre-laying group^67^.

AdipoQ has been originally identified as a protein secreted and expressed exclusively in adipose tissue^68–70^. AdipoQ showed many functions like expanding fatty acids oxidation, controlling glucose level and managing receptor activity. In humans, Adiponectin is known to stimulate the expression of *FABP*^16^. In chickens, adiponectin plays important roles in energy homeostasis, body weight, lipid metabolism, and insulin sensitivity^71–73^. In broiler chickens, Tahmoorespur et al.^74^ showed that *AdipoQ* expression in adipose tissue was inversely related to chicken abdominal fat deposition levels. Adiponectin has an effect on the impairment of adipocyte differentiation, which contributes to the negative regulation of fat deposition in chicken^71^. In adipose tissue of adult chickens, *AdipoQ* expression is higher in females than males, but *AdipoR1* expression was higher in males than females^17^. In female birds, Adiponectin is secreted into the blood from adipocytes with a higher serum level^75^. In emus, *eAdipoQ* expression did not show any seasonal (April to November) nor sexual variation. Emu fat gain from June to August regressed positively on June *eAdipoQ* expression but fat gain from August to November regressed negatively on August *eAdipoQ* expression. Similarly, fat gain from June to August also regressed positively on June *eAdipoR1* expression while fat gain from August to November regressed negatively on August *eAdipoR2* expression. White-throated sparrows increase fat deposits during pre-migratory periods and rely on these fat stores to fuel migration. In the adipose tissue, there was a significant change in the biological control of adipokine expression from pre-migratory conditions to migratory conditions. It was proposed that Adiponectin may play a role in the switch from fat deposition to lipid metabolism as the main source of energy to fuel migratory flight in birds^76^. In emus, *eAdipoR1/R2* expression was highest in April, before the birds started gaining fat. *eAdipoR1/R2* expression declined until the lowest level in November. Interestingly, in the oil extracted from emu fat in November, the % of Palmitic Acid (C16:0) regressed negatively on November *eAdipoR1* expression. Both Lenoleic Acid (C18:2n6) and α-linolenic acid (C18:3n3) regressed negatively on November *eAdipoR2* expression. These observations support the role of Adiponectin in lipid metabolism for converting the stored fat to energy during the period of low feed intake^50,51^. In emus, back fat showed a higher level of protein, cholesterol, C16:1 and the elements K, P, Si, Na, Ca, Mg, Fe, Zn, Se and Cu than abdominal fat. Abdominal fat was characterized by higher content of fat and ash, as well as Mn and Ba. Regardless of back or abdominal fat, there was generally high content of MUFA and PUFA. Males have higher content of Si, Ca, Cu, Sr in the adipose tissue than female^77^. In chickens, the most promising candidate genes affecting polyunsaturated fatty acids percentage were *FADS2*, *DCN*, *FRZB*, *OGN*, *PRKAG3*, *LHFP*, *CHCHD10*, *CYTL1*, *FBLN5*, and *ADGRD1*^78^.

There are two major methods of quantitative trait loci (QTL) determination, the candidate gene approach and the whole-genome scanning. The candidate gene approach is used to detect QTL (Quantitative Trait Loci) responsible for genetic variation in the traits of interest. In chickens, *FABP4* gene polymorphism has been associated with abdominal fat weight and percentage of abdominal fat, and *FABP4* gene could be a candidate locus or linked to a major gene(s) that affects abdominal fat content^79^. In pigs, *SCD-1* expression plays a critical role in adipocyte differentiation and has been identified as the promising candidate gene for less back fat deposition^80,81^. Fat deposition in emu is seasonal^77^. Under natural conditions, increased fat deposition in birds is for energy storage to cope with migration or periods when food is scarce, and is not associated with obesity. In addition to being an energy storage organ, the adipose is also an endocrine organ influencing reproduction, feeding behaviour, insulin sensitivity and disease resistance^75^. Whether genetic selection for increased fat deposition would lead to complications with obesity must be considered. In broiler chickens and turkeys, the selection for fast growth rate inevitably led to increase abdominal fat deposition and drastic reduction in breeder fertility^82,83^. Additionally, one has to consider whether selection for increased fat deposition would alter the fatty acids composition and other bioactive ingredients in the adipose tissue and thus affecting the efficacy of the emu oil^5^.

## Conclusion

There has not been any reported genetic selection studies for increasing subcutaneous fat deposition in farm animals and no genetic improvement of emu productive traits has been carried out due to their short history of domestication compared to other livestock species^84^. Our study has laid down the groundwork for identifying promising candidate genes for such purpose. A follow-up whole-genome scanning study^85^ explored the gene networks in emu adipose tissue affecting fat deposition and utilization and identified marker genes that deserve further analyses to develop novel molecular markers that can be applied to improve fat production in emus^86,87^.

## Supplementary Information

Supplementary Figure 1.

Supplementary Figure 2.

Supplementary Table S1.

Supplementary Table S2.

## Data Availability

The full-length cDNA of e*FABP4*, e*SCD1*, e*AdipoQ*, e*AdpoiR1*, e*AdipoR2*, e*LepR*, and *eβ-actin* were assigned the following GenBank accession numbers: *eFABP4* (JN663389), *eSCD1* (JN663390), *eAdipoQ* (JQ289558.1), *eAdipoR1* (JQ289559.1), *eAdipoR2* (JQ289560.1), *eLepR* (JQ289561), and housekeeping gene *eβ-actin* (JN663391). The primary protein structure assigned the nomenclature and Genbank accession numbers are: eFABP-4 (AET74082), eSCD-1 (AET74083), eAdipoQ (AFF19461), eAdopoR1 (AFF19462), eAdipoR2 (AFF19463), eLepR (AFF19464) and eβ-actin (AET74084). The datasets generated during and/or analysed during the current study are available from the corresponding author on reasonable request.
