# Supplementary material for: Seasonal and sexual variation in mRNA expression of selected adipokine genes affecting fat deposition and metabolism of the emu (Dromaius novaehollandiae)
Source: Sci Rep. 2022 Apr 15;12:6325. doi: 10.1038/s41598-022-10232-w (PMC9012844; doi:10.1038/s41598-022-10232-w)
Supplement: Supplementary file 1 — Supplementary Figure 1. [file 41598_2022_10232_MOESM1_ESM.docx]

**Seasonal and sexual variation in mRNA expression of selected adipokine genes affecting fat deposition and metabolism of the Emu (*Dromaius novaehollandiae*)**

**Supplemental Figure 1. Association of fat gain (kg) from April to June, from June to August, and from August to November, with mRNA expression level. (A). *eFABP4*. (B). *SCD1*. (C) *eAdipoQ*. (D) *AdipoR1*. (E). *AdipoR2*. (F) *eLepr.***

**April to June** **June to August** **August to November**

1. ***eFABP4***

R^2^= 0.712

P=0.0015

R^2^= 0.94

P=0.015

R^2^= 0.64

1. ***eSCD1***

P=0.76

P=0.0004

R^2^= 0.97

P=0.16

1. ***eAdipoQ***

R^2^= 0.829

R^2^= 0.86

1. ***eAdipoR1***

P=0.72

P=0.023

P=0.0103

R^2^= 0.92

R^2^= 0.55

1. ***eAdipoR2***

P=0.68

P=0.0018

P=0.78

R^2^= 0.825

1. ***eLepR***

P=0.2017

P=0.0026

R^2^= 0.92
