# Supplementary material for: Seasonal and sexual variation in mRNA expression of selected adipokine genes affecting fat deposition and metabolism of the emu (Dromaius novaehollandiae)
Source: Sci Rep. 2022 Apr 15;12:6325. doi: 10.1038/s41598-022-10232-w (PMC9012844; doi:10.1038/s41598-022-10232-w)
Supplement: Supplementary file 2 — Supplementary Figure 2. [file 41598_2022_10232_MOESM2_ESM.docx]

**Seasonal and sexual variation in mRNA expression of selected adipokine genes affecting fat deposition and metabolism of the Emu (*Dromaius novaehollandiae*)**

**Supplemental Figure 2A. Regression of (1) % Palmitic Acid (C16:0) and (2) % Oleic Acid (C18:1n-9) on Sex.**

1. **(2)**

R^2^= 0.19

R^2^= 0.18

| **Level** | **Least Sq Mean** | **Std Error** | **Mean** |
| --- | --- | --- | --- |
| **Female** | **25.972000** | **0.40164267** | **25.9720** |
| **Male** | **24.784286** | **0.33945001** | **24.7843** |

| **Level** | **Least Sq Mean** | **Std Error** | **Mean** |
| --- | --- | --- | --- |
| **Female** | **51.893000** | **0.59653225** | **51.8930** |
| **Male** | **53.615000** | **0.50416177** | **53.6150** |

**Supplemental Figure 2B**. **Regression of % Palmitic Acid (C16:0) in November emu oil to November *AdipoR1* expression level.**

R^2^= 0.48

**Supplemental Figure 2C**. **Regression of % Lenoleic Acid (C 8: 2n-6) in November emu oil to November *AdipoR2* expression level.**

R^2^= 0.54

**Supplemental Figure 2D**. **Regression of % α-Linolenic Acid (C18: 3n-3) in November emu oil to November *AdipoR2* expression level.**

R^2^= 0.52
