# Supplementary material for: Seasonal and sexual variation in mRNA expression of selected adipokine genes affecting fat deposition and metabolism of the emu (Dromaius novaehollandiae)
Source: Sci Rep. 2022 Apr 15;12:6325. doi: 10.1038/s41598-022-10232-w (PMC9012844; doi:10.1038/s41598-022-10232-w)
Supplement: Supplementary file 3 — Supplementary Table S1. [file 41598_2022_10232_MOESM3_ESM.docx]

**Kim et al. Seasonal and sexual variation in mRNA expression of selected adipokine genes affecting fat deposition and metabolism of the Emu (*Dromaius novaehollandiae*)**

**Supplemental Table S1**

**Schedule for Fat samples* collection**

|  | TagID | Sex | April | June | August | November |
| --- | --- | --- | --- | --- | --- | --- |
| 1 | R18 | F | X | X | X | X |
| 2 | R19 | M | X | X | X | X |
| 3 | R20 | F | X | X | X | X |
| 4 | R21 | F | X | X | X | X |
| 5 | R22 | F | X | X | X | X |
| 6 | Y10 | M | X | X | X | X |
| 7 | Y11 | M | X | X | X | X |
| 8 | Y12 | M | X | X | X | X |
| 9 | Y17 | M | X | X | X | X |
| 10 | Y18 | M | X | X | X | X |
| 11 | Y20 | M | X | X | X | X |
| 12 | 11No | M |  |  |  | X |
| 13 | 11R16 | F |  |  |  | X |
| 14 | 11R5 | F |  |  |  | X |
| 15 | 11Y13 | M |  |  |  | X |
| 16 | 11Y15 | M |  |  |  | X |
| 17 | 11Y16 | M |  |  |  | X |
| 18 | 6AAA | F |  | X |  |  |
| 19 | 6BBB | M |  | X |  |  |
| 20 | 6DFA | F |  | X |  | **X** |
| 21 | 6R9 | F |  | X |  |  |
| 22 | 6Y21 | M |  | X |  | **X** |
| 23 | 6Y22 | M |  | X |  | **X** |
| 24 | 8R17 | F |  |  | X | **X** |
| 25 | 8R23 | F |  |  | X |  |
| 26 | 8R24 | F |  |  | X | **X** |
| 27 | 8Y23 | M |  |  | X | **X** |
| 28 | 8Y24 | M |  |  | X |  |
| 29 | 8Y25 | M |  |  | X |  |

**X Additional fat samples collected in November for fatty acids analysis**

***For seasonal gene expression level analysis, N=62; For association of gene expression level with fat gain, N =44; For Fatty Acids analysis, N= 24**
