# Supplementary material for: Seasonal and sexual variation in mRNA expression of selected adipokine genes affecting fat deposition and metabolism of the emu (Dromaius novaehollandiae)
Source: Sci Rep. 2022 Apr 15;12:6325. doi: 10.1038/s41598-022-10232-w (PMC9012844; doi:10.1038/s41598-022-10232-w)
Supplement: Supplementary file 4 — Supplementary Table S2. [file 41598_2022_10232_MOESM4_ESM.docx]

**Kim et al. Seasonal and sexual variation in mRNA expression of selected adipokine genes affecting fat deposition and metabolism of the Emu (*Dromaius novaehollandiae*)**

**Supplemental Table S2**

1. **Response FABP**

**Whole Model**

**Summary of Fit**

|  |  |
| --- | --- |
| RSquare | 0.375032 |
| RSquare Adj | 0.294018 |
| Root Mean Square Error | 0.650111 |
| Mean of Response | 0.931606 |
| Observations (or Sum Wgts) | 62 |

**REML Variance Component Estimates**

| **Random Effect** | **Var Ratio** | **Var Component** | **Std Error** | **95% Lower** | **95% Upper** | **Pct of Total** |
| --- | --- | --- | --- | --- | --- | --- |
| ID[Sex] | 0.0034529 | 0.0014594 | 0.0534119 | -0.103228 | 0.1061466 | 0.344 |
| ID*Season[Sex] |  | 0.4226441 | 0.0971039 | 0.2821296 | 0.7025822 | 99.656 |
| Total |  | 0.4241034 |  |  |  | 100.000 |

**Fixed Effect Tests**

| **Source** | **Nparm** | **DF** | **DFDen** | **F Ratio** | **Prob > F** |  |
| --- | --- | --- | --- | --- | --- | --- |
| Sex | 1 | 1 | 18.55 | 0.7789 | 0.3888 |  |
| Season | 3 | 3 | 44.76 | 5.1165 | 0.0040* |  |
| Sex*Season | 3 | 3 | 44.76 | 2.5780 | 0.0654 |  |

**Effect Details**

**Sex**

**Least Squares Means Table**

| **Level** | **Least Sq Mean** |  | **Std Error** |
| --- | --- | --- | --- |
| F | 0.83665826 |  | 0.14129278 |
| M | 0.99500239 |  | 0.11057644 |

**Season**

**Least Squares Means Table**

| **Level** | **Least Sq Mean** |  | **Std Error** |
| --- | --- | --- | --- |
| April | 1.0591968 |  | 0.20761797 |
| June | 0.6370185 |  | 0.16159541 |
| August | 0.5885163 |  | 0.16159541 |
| November | 1.3785897 |  | 0.16656769 |

**LSMeans Differences Tukey HSD**

α=

0.050

LSMean[i] By LSMean[j]

| Mean[i]-Mean[j]  Std Err Dif  Lower CL Dif  Upper CL Dif | April | June | August | November |
| --- | --- | --- | --- | --- |
| April | 0  0  0  0 | 0.42218  0.26052  -0.2739  1.11824 | 0.47068  0.26052  -0.2254  1.16674 | -0.3194  0.26333  -1.0234  0.38459 |
| June | -0.4222  0.26052  -1.1182  0.27388 | 0  0  0  0 | 0.0485  0.22752  -0.5578  0.65478 | -0.7416  0.23096  -1.3571  -0.126 |
| August | -0.4707  0.26052  -1.1667  0.22538 | -0.0485  0.22752  -0.6548  0.55778 | 0  0  0  0 | -0.7901  0.23096  -1.4056  -0.1745 |
| November | 0.31939  0.26333  -0.3846  1.02337 | 0.74157  0.23096  0.12601  1.35713 | 0.79007  0.23096  0.17451  1.40564 | 0  0  0  0 |

| **Level** |  |  | **Least Sq Mean** |
| --- | --- | --- | --- |
| November | A |  | 1.3785897 |
| April | A | B | 1.0591968 |
| June |  | B | 0.6370185 |
| August |  | B | 0.5885163 |

Levels not connected by same letter are significantly different.

**Sex*Season**

**Least Squares Means Table**

| **Level** | **Least Sq Mean** |  | **Std Error** |
| --- | --- | --- | --- |
| F,April | 1.0022365 |  | 0.33165592 |
| F,June | 0.6954549 |  | 0.24782932 |
| F,August | 0.7263965 |  | 0.24782932 |
| F,November | 0.9225452 |  | 0.26825172 |
| M,April | 1.1161572 |  | 0.24985042 |
| M,June | 0.5785821 |  | 0.20744380 |
| M,August | 0.4506361 |  | 0.20744380 |
| M,November | 1.8346342 |  | 0.19753529 |

**(B) Response SCD**

**Summary of Fit**

|  |  |
| --- | --- |
| RSquare | -1.30561 |
| RSquare Adj | -1.60448 |
| Root Mean Square Error | 0.256808 |
| Mean of Response | 0.093399 |
| Observations (or Sum Wgts) | 62 |

**REML Variance Component Estimates**

| **Random Effect** | **Var Ratio** | **Var Component** | **Std Error** | **95% Lower** | **95% Upper** | **Pct of Total** |
| --- | --- | --- | --- | --- | --- | --- |
| ID[Sex] | -0.216021 | -0.014247 | 0.0040505 | -0.022186 | -0.006308 | -27.554 |
| ID*Season[Sex] |  | 0.0659506 | 0.0146898 | 0.044516 | 0.1077379 | 127.554 |
| Total |  | 0.0517039 |  |  |  | 100.000 |

**Fixed Effect Tests**

| **Source** | **Nparm** | **DF** | **DFDen** | **F Ratio** | **Prob > F** |  |
| --- | --- | --- | --- | --- | --- | --- |
| Sex | 1 | 1 | 7.77 | 2.4067 | 0.1605 |  |
| Season | 3 | 3 | 42.33 | 2.9858 | 0.0417* |  |
| Sex*Season | 3 | 3 | 42.33 | 3.0016 | 0.0410* |  |

**Effect Details**

**Sex**

**Least Squares Means Table**

| **Level** | **Least Sq Mean** |  | **Std Error** |
| --- | --- | --- | --- |
| F | 0.04825760 |  | 0.02337002 |
| M | 0.09376283 |  | 0.01772668 |

**Season**

**Least Squares Means Table**

| **Level** | **Least Sq Mean** |  | **Std Error** |
| --- | --- | --- | --- |
| April | 0.0736517 |  | 0.06757918 |
| June | 0.0072533 |  | 0.05498395 |
| August | 0.2178357 |  | 0.05498395 |
| November | -0.0146998 |  | 0.05647329 |

**Sex*Season**

**Least Squares Means Table**

| **Level** | **Least Sq Mean** |  | **Std Error** |
| --- | --- | --- | --- |
| F,April | -0.0821188 |  | 0.10727492 |
| F,June | -0.0212855 |  | 0.08437009 |
| F,August | 0.3460059 |  | 0.08437009 |
| F,November | -0.0495712 |  | 0.09056406 |
| M,April | 0.2294222 |  | 0.08221846 |
| M,June | 0.0357921 |  | 0.07053104 |
| M,August | 0.0896654 |  | 0.07053104 |
| M,November | 0.0201717 |  | 0.06749133 |

**LSMeans Differences Tukey HSD**

α=

0.050

LSMean[i] By LSMean[j]

| Mean[i]-Mean[j]  Std Err Dif  Lower CL Dif  Upper CL Dif | F,April | F,June | F,August | F,November | M,April | M,June | M,August | M,November |
| --- | --- | --- | --- | --- | --- | --- | --- | --- |
| F,April | 0  0  0  0 | -0.0608  0.15407  -0.3716  0.24993 | -0.4281  0.15407  -0.7389  -0.1174 | -0.0325  0.16066  -0.3568  0.29167 | -0.3115  0.13516  -0.583  -0.0401 | -0.1179  0.12838  -0.3758  0.13999 | -0.1718  0.12838  -0.4297  0.08612 | -0.1023  0.12674  -0.3569  0.15231 |
| F,June | 0.06083  0.15407  -0.2499  0.3716 | 0  0  0  0 | -0.3673  0.12984  -0.6292  -0.1054 | 0.02829  0.13583  -0.2457  0.30231 | -0.2507  0.11781  -0.4875  -0.0139 | -0.0571  0.10997  -0.2783  0.16416 | -0.111  0.10997  -0.3322  0.11028 | -0.0415  0.10804  -0.2588  0.17592 |
| F,August | 0.42812  0.15407  0.11736  0.73889 | 0.36729  0.12984  0.10539  0.6292 | 0  0  0  0 | 0.39558  0.13583  0.12155  0.6696 | 0.11658  0.11781  -0.1203  0.35343 | 0.31021  0.10997  0.08898  0.53145 | 0.25634  0.10997  0.03511  0.47758 | 0.32583  0.10804  0.10845  0.54321 |
| F,November | 0.03255  0.16066  -0.2917  0.35677 | -0.0283  0.13583  -0.3023  0.24574 | -0.3956  0.13583  -0.6696  -0.1216 | 0  0  0  0 | -0.279  0.12232  -0.5249  -0.0331 | -0.0854  0.11479  -0.3162  0.14552 | -0.1392  0.11479  -0.3701  0.09165 | -0.0697  0.11295  -0.2969  0.15745 |
| M,April | 0.31154  0.13516  0.04011  0.58298 | 0.25071  0.11781  0.01387  0.48755 | -0.1166  0.11781  -0.3534  0.12026 | 0.27899  0.12232  0.03311  0.52488 | 0  0  0  0 | 0.19363  0.12323  -0.0551  0.44238 | 0.13976  0.12323  -0.109  0.38851 | 0.20925  0.12002  -0.0329  0.45144 |
| M,June | 0.11791  0.12838  -0.14  0.37581 | 0.05708  0.10997  -0.1642  0.27831 | -0.3102  0.10997  -0.5314  -0.089 | 0.08536  0.11479  -0.1455  0.31625 | -0.1936  0.12323  -0.4424  0.05512 | 0  0  0  0 | -0.0539  0.11039  -0.2767  0.16891 | 0.01562  0.10739  -0.2011  0.23231 |
| M,August | 0.17178  0.12838  -0.0861  0.42969 | 0.11095  0.10997  -0.1103  0.33219 | -0.2563  0.10997  -0.4776  -0.0351 | 0.13924  0.11479  -0.0916  0.37012 | -0.1398  0.12323  -0.3885  0.10899 | 0.05387  0.11039  -0.1689  0.27665 | 0  0  0  0 | 0.06949  0.10739  -0.1472  0.28618 |
| M,November | 0.10229  0.12674  -0.1523  0.35689 | 0.04146  0.10804  -0.1759  0.25884 | -0.3258  0.10804  -0.5432  -0.1085 | 0.06974  0.11295  -0.1574  0.29694 | -0.2093  0.12002  -0.4514  0.03294 | -0.0156  0.10739  -0.2323  0.20107 | -0.0695  0.10739  -0.2862  0.14719 | 0  0  0  0 |

| **Level** |  |  |  | **Least Sq Mean** |
| --- | --- | --- | --- | --- |
| F,August | A |  |  | 0.3460059 |
| M,April | A | B |  | 0.2294222 |
| M,August |  | B | C | 0.0896654 |
| M,June |  | B | C | 0.0357921 |
| M,November |  | B | C | 0.0201717 |
| F,June |  |  | C | -0.0212855 |
| F,November |  |  | C | -0.0495712 |
| F,April |  |  | C | -0.0821188 |

Levels not connected by same letter are significantly different.

**(C) Response AQ**

**Whole Model**

**Summary of Fit**

|  |  |
| --- | --- |
| RSquare | 0.961907 |
| RSquare Adj | 0.956969 |
| Root Mean Square Error | 0.00563 |
| Mean of Response | 0.019646 |
| Observations (or Sum Wgts) | 62 |

**REML Variance Component Estimates**

| **Random Effect** | **Var Ratio** | **Var Component** | **Std Error** | **95% Lower** | **95% Upper** | **Pct of Total** |
| --- | --- | --- | --- | --- | --- | --- |
| Individual[Sex] | 12.622068 | 0.0004001 | 0.0001185 | 0.0001677 | 0.0006324 | 92.659 |
| Residual |  | 0.0000317 | 8.8035e-6 | 1.9645e-5 | 5.959e-5 | 7.341 |
| Total |  | 0.0004318 |  |  |  | 100.000 |

**Fixed Effect Tests**

| **Source** | **Nparm** | **DF** | **DFDen** | **F Ratio** | **Prob > F** |  |
| --- | --- | --- | --- | --- | --- | --- |
| Sex | 1 | 1 | 25.98 | 2.9266 | 0.0990 |  |
| Month | 3 | 3 | 27.51 | 1.5368 | 0.2272 |  |
| Month*Sex | 3 | 3 | 27.51 | 0.4981 | 0.6866 |  |

**Effects Details**

**Sex**

**Least Squares Means Table**

| **Level** | **Least Sq Mean** |  | **Std Error** |
| --- | --- | --- | --- |
| F | 0.03336337 |  | 0.00596844 |
| M | 0.02004965 |  | 0.00499447 |

**Season**

**Least Squares Means Table**

| **Level** | **Least Sq Mean** |  | **Std Error** |
| --- | --- | --- | --- |
| APRIL | 0.02554219 |  | 0.00429498 |
| AUG | 0.02959773 |  | 0.00412593 |
| JUNE | 0.02702142 |  | 0.00412593 |
| NOV | 0.02466470 |  | 0.00414955 |

**Month*Sex**

**Least Squares Means Table**

| **Level** | **Least Sq Mean** |  | **Std Error** |
| --- | --- | --- | --- |
| APRIL,F | 0.03055190 |  | 0.00664439 |
| APRIL,M | 0.02053248 |  | 0.00544422 |
| AUG,F | 0.03757885 |  | 0.00633146 |
| AUG,M | 0.02161661 |  | 0.00529205 |
| JUNE,F | 0.03362685 |  | 0.00633146 |
| JUNE,M | 0.02041600 |  | 0.00529205 |
| NOV,F | 0.03169588 |  | 0.00643358 |
| NOV,M | 0.01763352 |  | 0.00524254 |

**(D) Response AR1**

**Summary of Fit**

|  |  |
| --- | --- |
| RSquare | 0.349665 |
| RSquare Adj | 0.265362 |
| Root Mean Square Error | 0.000816 |
| Mean of Response | 0.002426 |
| Observations (or Sum Wgts) | 62 |

**REML Variance Component Estimates**

| **Random Effect** | **Var Ratio** | **Var Component** | **Std Error** | **95% Lower** | **95% Upper** | **Pct of Total** |
| --- | --- | --- | --- | --- | --- | --- |
| ID[Sex] | 0.1219857 | 8.1159e-8 | 1.0565e-7 | -1.259e-7 | 2.8823e-7 | 10.872 |
| ID*Season[Sex] |  | 6.6532e-7 | 1.5387e-7 | 4.4306e-7 | 1.1102e-6 | 89.128 |
| Total |  | 7.4648e-7 |  |  |  | 100.000 |

**Fixed Effect Tests**

| **Source** | **Nparm** | **DF** | **DFDen** | **F Ratio** | **Prob > F** |  |
| --- | --- | --- | --- | --- | --- | --- |
| Sex | 1 | 1 | 22.87 | 1.0566 | 0.3147 |  |
| Season | 3 | 3 | 44.84 | 2.6043 | 0.0505* |  |
| Sex*Season | 3 | 3 | 44.84 | 0.9352 | 0.4316 |  |

**Effect Details**

**Sex**

**Least Squares Means Table**

| **Level** | **Least Sq Mean** |  | **Std Error** |
| --- | --- | --- | --- |
| F | 0.00265144 |  | 0.00019844 |
| M | 0.00239104 |  | 0.00015746 |

**ID[Sex]**

**Season**

**Least Squares Means Table**

| **Level** | **Least Sq Mean** |  | **Std Error** |
| --- | --- | --- | --- |
| April | 0.00310916 |  | 0.00026912 |
| June | 0.00230200 |  | 0.00021234 |
| August | 0.00239813 |  | 0.00021234 |
| November | 0.00227567 |  | 0.00021862 |

**LSMeans Differences Student's t**

α=

0.050

LSMean[i] By LSMean[j]

| Mean[i]-Mean[j]  Std Err Dif  Lower CL Dif  Upper CL Dif | April | June | August | November |
| --- | --- | --- | --- | --- |
| April | 0  0  0  0 | 0.00081  0.00033  0.00014  0.00147 | 0.00071  0.00033  4.71e-5  0.00138 | 0.00083  0.00033  0.00016  0.0015 |
| June | -0.0008  0.00033  -0.0015  -0.0001 | 0  0  0  0 | -0.0001  0.00029  -0.0007  0.00049 | 2.63e-5  0.00029  -0.0006  0.00062 |
| August | -0.0007  0.00033  -0.0014  -4.7e-5 | 0.0001  0.00029  -0.0005  0.00068 | 0  0  0  0 | 0.00012  0.00029  -0.0005  0.00071 |
| November | -0.0008  0.00033  -0.0015  -0.0002 | -2.6e-5  0.00029  -0.0006  0.00057 | -0.0001  0.00029  -0.0007  0.00047 | 0  0  0  0 |

| **Level** |  |  | **Least Sq Mean** |
| --- | --- | --- | --- |
| April | A |  | 0.00310916 |
| August |  | B | 0.00239813 |
| June |  | B | 0.00230200 |
| November |  | B | 0.00227567 |

Levels not connected by same letter are significantly different.

**Sex*Season**

**Least Squares Means Table**

| **Level** | **Least Sq Mean** |  | **Std Error** |
| --- | --- | --- | --- |
| F,April | 0.00298941 |  | 0.00042919 |
| F,June | 0.00255228 |  | 0.00032575 |
| F,August | 0.00276499 |  | 0.00032575 |
| F,November | 0.00229906 |  | 0.00035158 |
| M,April | 0.00322892 |  | 0.00032481 |
| M,June | 0.00205171 |  | 0.00027249 |
| M,August | 0.00203126 |  | 0.00027249 |
| M,November | 0.00225228 |  | 0.00025993 |

**(E) Response AR2**

**Summary of Fit**

|  |  |
| --- | --- |
| RSquare | 0.667318 |
| RSquare Adj | 0.624192 |
| Root Mean Square Error | 0.000539 |
| Mean of Response | 0.0009 |
| Observations (or Sum Wgts) | 62 |

**REML Variance Component Estimates**

| **Random Effect** | **Var Ratio** | **Var Component** | **Std Error** | **95% Lower** | **95% Upper** | **Pct of Total** |
| --- | --- | --- | --- | --- | --- | --- |
| ID[Sex] | 0.313509 | 9.0925e-8 | 6.0919e-8 | -2.848e-8 | 2.1033e-7 | 23.868 |
| ID*Season[Sex] |  | 2.9002e-7 | 6.7762e-8 | 1.9242e-7 | 4.8684e-7 | 76.132 |
| Total |  | 3.8095e-7 |  |  |  | 100.000 |

**Fixed Effect Tests**

| **Source** | **Nparm** | **DF** | **DFDen** | **F Ratio** | **Prob > F** |  |
| --- | --- | --- | --- | --- | --- | --- |
| Sex | 1 | 1 | 27.14 | 1.9943 | 0.1693 |  |
| Season | 3 | 3 | 44.36 | 19.3662 | <.0001* |  |
| Sex*Season | 3 | 3 | 44.36 | 1.7716 | 0.1664 |  |

**Effect Details**

**Sex**

**Least Squares Means Table**

| **Level** | **Least Sq Mean** |  | **Std Error** |
| --- | --- | --- | --- |
| F | 0.00119450 |  | 0.00015262 |
| M | 0.00091825 |  | 0.00012236 |

**Season**

**Least Squares Means Table**

| **Level** | **Least Sq Mean** |  | **Std Error** |
| --- | --- | --- | --- |
| April | 0.00206979 |  | 0.00018817 |
| June | 0.00071574 |  | 0.00015026 |
| August | 0.00098783 |  | 0.00015026 |
| November | 0.00045214 |  | 0.00015452 |

**LSMeans Differences Tukey HSD**

α=

0.050

LSMean[i] By LSMean[j]

| Mean[i]-Mean[j]  Std Err Dif  Lower CL Dif  Upper CL Dif | April | June | August | November |
| --- | --- | --- | --- | --- |
| April | 0  0  0  0 | 0.00135  0.00022  0.00076  0.00194 | 0.00108  0.00022  0.00049  0.00167 | 0.00162  0.00022  0.00102  0.00221 |
| June | -0.0014  0.00022  -0.0019  -0.0008 | 0  0  0  0 | -0.0003  0.0002  -0.0008  0.00025 | 0.00026  0.0002  -0.0003  0.00079 |
| August | -0.0011  0.00022  -0.0017  -0.0005 | 0.00027  0.0002  -0.0003  0.0008 | 0  0  0  0 | 0.00054  0.0002  4.62e-6  0.00107 |
| November | -0.0016  0.00022  -0.0022  -0.001 | -0.0003  0.0002  -0.0008  0.00027 | -0.0005  0.0002  -0.0011  -4.6e-6 | 0  0  0  0 |

| **Level** |  |  |  | **Least Sq Mean** |
| --- | --- | --- | --- | --- |
| April | A |  |  | 0.00206979 |
| August |  | B |  | 0.00098783 |
| June |  | B | C | 0.00071574 |
| November |  |  | C | 0.00045214 |

Levels not connected by same letter are significantly different.

**Sex*Season**

**Least Squares Means Table**

| **Level** | **Least Sq Mean** |  | **Std Error** |
| --- | --- | --- | --- |
| F,April | 0.00250671 |  | 0.00029967 |
| F,June | 0.00069288 |  | 0.00023057 |
| F,August | 0.00113302 |  | 0.00023057 |
| F,November | 0.00044540 |  | 0.00024817 |
| M,April | 0.00163286 |  | 0.00022766 |
| M,June | 0.00073861 |  | 0.00019274 |
| M,August | 0.00084264 |  | 0.00019274 |
| M,November | 0.00045887 |  | 0.00018417 |

**(F) Response LepR**

**Summary of Fit**

|  |  |
| --- | --- |
| RSquare | 0.656113 |
| RSquare Adj | 0.611535 |
| Root Mean Square Error | 9.514e-5 |
| Mean of Response | 0.000263 |
| Observations (or Sum Wgts) | 62 |

**REML Variance Component Estimates**

| **Random Effect** | **Var Ratio** | **Var Component** | **Std Error** | **95% Lower** | **95% Upper** | **Pct of Total** |
| --- | --- | --- | --- | --- | --- | --- |
| ID[Sex] | 0.6601348 | 5.9753e-9 | 3.1641e-9 | -2.26e-10 | 1.2177e-8 | 39.764 |
| ID*Season[Sex] |  | 9.0516e-9 | 2.2961e-9 | 5.8206e-9 | 1.5985e-8 | 60.236 |
| Total |  | 1.5027e-8 |  |  |  | 100.000 |

**Fixed Effect Tests**

| **Source** | **Nparm** | **DF** | **DFDen** | **F Ratio** | **Prob > F** |  |
| --- | --- | --- | --- | --- | --- | --- |
| Sex | 1 | 1 | 25.83 | 0.2576 | 0.6161 |  |
| Season | 3 | 3 | 39.64 | 4.9077 | 0.0054* |  |
| Sex*Season | 3 | 3 | 39.64 | 1.8034 | 0.1622 |  |

**Effect Details**

**Sex**

**Least Squares Means Table**

| **Level** | **Least Sq Mean** |  | **Std Error** |
| --- | --- | --- | --- |
| F | 0.00026531 |  | 0.00003219 |
| M | 0.00028634 |  | 0.00002610 |

**ID[Sex]**

**Season**

**Least Squares Means Table**

| **Level** | **Least Sq Mean** |  | **Std Error** |
| --- | --- | --- | --- |
| April | 0.00035008 |  | 0.00003572 |
| June | 0.00027999 |  | 0.00002922 |
| August | 0.00027441 |  | 0.00002922 |
| November | 0.00019881 |  | 0.00002997 |

**LSMeans Differences Tukey HSD**

α=

0.050

LSMean[i] By LSMean[j]

| Mean[i]-Mean[j]  Std Err Dif  Lower CL Dif  Upper CL Dif | April | June | August | November |
| --- | --- | --- | --- | --- |
| April | 0  0  0  0 | 0.00007  0.00004  -3.6e-5  0.00018 | 7.57e-5  0.00004  -3.1e-5  0.00018 | 0.00015  0.00004  4.4e-5  0.00026 |
| June | -0.0001  0.00004  -0.0002  3.64e-5 | 0  0  0  0 | 5.57e-6  3.6e-5  -0.0001  0.0001 | 8.12e-5  3.64e-5  -1.6e-5  0.00018 |
| August | -0.0001  0.00004  -0.0002  0.00003 | -5.6e-6  3.6e-5  -0.0001  0.00009 | 0  0  0  0 | 7.56e-5  3.64e-5  -2.2e-5  0.00017 |
| November | -0.0002  0.00004  -0.0003  -4.4e-5 | -0.0001  3.64e-5  -0.0002  1.61e-5 | -0.0001  3.64e-5  -0.0002  2.16e-5 | 0  0  0  0 |

| **Level** |  |  | **Least Sq Mean** |
| --- | --- | --- | --- |
| April | A |  | 0.00035008 |
| June | A | B | 0.00027999 |
| August | A | B | 0.00027441 |
| November |  | B | 0.00019881 |

Levels not connected by same letter are significantly different.

**Sex*Season**

**Least Squares Means Table**

| **Level** | **Least Sq Mean** |  | **Std Error** |
| --- | --- | --- | --- |
| F,April | 0.00028157 |  | 0.00005673 |
| F,June | 0.00028378 |  | 0.00004486 |
| F,August | 0.00029083 |  | 0.00004486 |
| F,November | 0.00020505 |  | 0.00004799 |
| M,April | 0.00041858 |  | 0.00004343 |
| M,June | 0.00027619 |  | 0.00003745 |
| M,August | 0.00025800 |  | 0.00003745 |
| M,November | 0.00019257 |  | 0.00003592 |
